# Affective Communication for Socially Assistive Robots (SARs) for Children with Autism Spectrum Disorder: A Systematic Review

**DOI:** 10.3390/s21155166

**Published:** 2021-07-30

**Authors:** Sandra Cano, Carina S. González, Rosa María Gil-Iranzo, Sergio Albiol-Pérez

**Affiliations:** 1 School of Computer Engineering, Pontificia Universidad Católica de Valparaíso, Valparaíso 2340000, Chile; 2 Department of Computer Engineering and Systems, University of La Laguna, 38204 La Laguna, Spain; cjgonza@ull.edu.es; 3 Department of Computer Engineering and Industrial, University of Lleida, 25001 Lleida, Spain; rgil@diei.udl.cat; 4 Aragón Health Research Institute (IIS Aragón), Universidad de Zaragoza, Cdad. Escolar, 4, 44003 Teruel, Spain; salbiol@unizar.es

**Keywords:** affective computing, autism spectrum disorders, affective human–robot interaction, socially assistive robots, therapeutic intervention

## Abstract

Research on affective communication for socially assistive robots has been conducted to enable physical robots to perceive, express, and respond emotionally. However, the use of affective computing in social robots has been limited, especially when social robots are designed for children, and especially those with autism spectrum disorder (ASD). Social robots are based on cognitive-affective models, which allow them to communicate with people following social behaviors and rules. However, interactions between a child and a robot may change or be different compared to those with an adult or when the child has an emotional deficit. In this study, we systematically reviewed studies related to computational models of emotions for children with ASD. We used the Scopus, WoS, Springer, and IEEE-Xplore databases to answer different research questions related to the definition, interaction, and design of computational models supported by theoretical psychology approaches from 1997 to 2021. Our review found 46 articles; not all the studies considered children or those with ASD.

## 1. Introduction

Robots are devices that use sensors to monitor human movement and positioning and then use this feedback to interact with the environment. With the use of sensors and actuators, robots are capable of measuring and storing patient function parameters that can aid long-term clinical evaluation. With the ability to detect and measure small changes in movements and forces, these devices can assist therapists in the processes of treatment planning and goal setting. However, the design of a physical robot does not have intelligence or affective behavior and cannot react to the user’s behavior. It cannot, therefore, establish a fluid interaction with the user. For this reason, robots require computational models that can provide these empathy skills.

Socially assistive robots (SARs) are designed to help people’s well-being and care, especially children with autism spectrum disorder (ASD). Tapus et al. [1] designed an intelligent cognitive model for SARs during therapy for people with dementia that integrates artificial intelligence and affective computing. Other researchers explored the user’s affective state as a mechanism to adapt the behavior of the robot [2], by which the robot learns appropriate behaviors by considering the physiological signals of children. Interactions between adults and children can vary widely, even more so when the child has ASD.

Those with ASD are at the highest risk of suffering complications as a result of anxiety, learning problems, immune system alterations, behavioral problems, impaired social communication, attention disorder with hyperactivity, irritability, and aggression. These conditions represent additional challenges during the COVID-19 pandemic [3]. According to the Diagnostic and Statistical Manual (DSM-5) [4], children with ASD have deficits in emotional communication in recognizing, understanding, and responding to emotional and mental states. Therefore, they have problems related to recognizing emotions from facial expressions, vocal intonation, body language, and physiological signals, as well as understanding emotions and how to respond emotionally when interacting with another person.

In 2002, Picard defined the term “affective computing” [5]. She stated that it is important to consider adapting a machine to the affective state of the user or their personality traits, hinting that many systems that have been created that focus on logical reasoning rather than emotional aspects. Therefore, a machine, to be affective, must perceive, interpret, process, and simulate human affects. Affective computing is an interdisciplinary field that incorporates computer science, psychology, and cognitive science.

To achieve this effective high level of interaction, the system must be endowed with intelligence. A socially intelligent robot must be capable of extracting information in real time from a social environment and respond according to human behavior. However, this social robot can respond without mimicking emotional responses, which can be defined as cognitive empathy. Emotional empathy refers to sensitivity and understanding the mental states of others. Therefore, intelligent emotional communication must provide artificial and emotional intelligence to the SARs. When robots interact with humans, emotions are essential for human social interactions; however, many studies are focused on specific abilities, such as expressing and/or recognizing emotions. Emotional empathy includes the ability to perceive, use, understand, and manage emotions. In addition, many of the proposed models are focused on virtual agents, without considering approaches to robotics, which may include types of sensors, appearance (mechanical characteristics), and control theories, which may affect the model. For example, Cathexis [6] is an emotional computational model that was designed initially on a virtual agent, and then was modified considering approaches to robotics, where it was implemented in a Yuppy robot [7] to determine needs such as temperature-, fatigue-, and curiosity-associated senses from different sensors. Thus, intelligent emotional communication may impact psychology, artificial intelligence, industrial control, and robotics.

Emotional communication for robotic systems focused on children with ASD should involve the robot being able to perceive, interpret, communicate, and adapt emotional states through social interactions [8]. The robot should require an affective detection system that recognizes if the child is experiencing positive or negative feelings, as well as reasoning that can be displayed at a cognitive level [9]. Research on social robots uses cognitive-affective architectures, which are usually modeled for the behavior of a person without a disability and with basic skills. This means that the empathy recognition of the robot is focused on interacting with a person without special needs. In 2020, a literature review on computational emotion models [10] found that several computational models of emotions have been proposed to enable artificial agents to generate emotions. However, specific barriers limit full capabilities in these models. In addition, several of the models proposed do not include emotions but are based more upon a cognitive approach. Many of the proposed computational models have not been used in robotic systems. However, virtual agents have been used when designing the affective computational model focused on children, especially those with ASD.

## 2. Background

This section introduces the basic concepts to understand the need to present this work.

### 2.1. Autism Spectrum Disorder (ASD)

Autism spectrum disorder is related to social and communication difficulties and low interest, as well as repetitive behaviors. According to the DSM-5, ASD is coded for three levels of performance. At level one, children need help because even though their vocabulary has not been affected, they express atypical or unsatisfactory responses to other people’s social openness. It may seem that these children have little interest in social interactions. At level two, the children need notable help, as they have conditions in verbal and nonverbal communication skills, limited social interactions, and restricted and inflexible behaviors that affect their performance. At level three, the children need very noticeable help, as severe deficits in verbal and nonverbal social communication skills cause severe disturbances in performance, very limited initiation of social interactions, and minimal responses to other people’s social openness.

Several typical social issues are demonstrated by children with ASD, with deficits related to, amongst others, a lack of direct eye gaze or eye contact [11]. One of the most prominent symptoms is quantitative and qualitative deficits in the social communication process, as well as a tendency toward isolation, only joining other children when obliged to do so. In addition, the child has deficiencies in verbal and non-verbal communication, difficulty in perceiving and understanding emotions, resistance to any change in the surrounding environment, and challenges in language development [12]. The models that have been proposed did not focus on these difficulties encountered in children with ASD, such as their deficits in the ability to make eye contact, interpret feelings, and understand tones of voice or facial expressions, amongst others.

These difficulties with emotion recognition and expression are related to the theory of mind (ToM) [13], which is defined as the ability to attribute mental states, beliefs, intents, desires, emotions, and knowledge to ourselves and others. Some children with ASD, however, have high functionalities and can recognize the emotions of facial expressions, but unlike a typical child, their identification process is different [14]. This may be because children with ASD process visual information differently than a typically developing child [15].

### 2.2. What Is an Emotion?

An emotion is a brief episode that occurs in the brain, producing autonomic and behavioral changes [16]. There are basic emotions that are considered to be innate and universal in all cultures. Basic emotions can exist in combinations to form other, more complex emotions. Ekman et al. [17] identified six primary or basic emotions in facial expressions: anger, fear, joy, sadness, surprise, and disgust. However, others consider that there are more than six primary emotions [18].

An emotion can be represented as a dimensional model [19] that includes aspects such as arousal, valence, and dominance. Arousal is the level of activation or emotional intensity. Valence defines whether it is a positive or negative emotion. Dominance is the degree of emotional control.

There is no unique definition of emotions. Different authors have tried to define emotions from different approaches. These approaches can be grouped into three: physiological, neurological, and cognitive. For instance, the James–Lange theory [20,21] features a physiological approach, contending that emotions occur as a result of physiological reactions to events. Another theory of emotion from a cognitive approach is that of Shachter and Singer [22], which proposes that emotions are composed of two factors: physiological and cognitive. This theory suggests that physiological arousal occurs first, and then the subject must identify the reason for this arousal to experience it and label it as an emotion. In other words, a stimulus leads to a physiological response that is then cognitively interpreted and labeled. This is also supported by the Cannon–Bard theory [23], which states that similar physiological responses can produce different emotions, for example, if the hands are sweating and the heart rate has increased, it is identified as an emotional state of anxiety. Lazarus’s theory [24] states that thinking must occur before experiencing an emotion. Different appraisal theories have been proposed, for example, by Frijda [25], Roseman [26], and Ortoney et al. [27]. Another approach is the neurological one, the theories of which, such as Damasio’s [28], explain the relationship between emotions and reason.

### 2.3. Emotional Intelligence

Emotional intelligence (EI) is defined as the ability to identify, evaluate, and regulate the emotions of oneself, others, and groups. It is the ability to perceive, understand, and use emotional information within an environment [29], but few studies have related EI to children with ASD. This is due in part because there are branches of psychology that do not contemplate this concept.

However, a priori, it seems that children with ASD have deficits in communicating, processing, and integrating information from the environment, and establishing and maintaining reciprocal social relationships, inferring interests from others, and transitioning to new learning environments [30] could be potential beneficiaries of a line of research associated with IE. Thus, one of the key processes of emotional intelligence is emotion regulation (ER). ER can be defined as the ability to monitor, evaluate, and modify one’s emotional state to achieve a goal [31].

Lieu et al. [32] proposed a model of cognitive-emotional regulation in human–robot interaction based on Gross’s emotional regulation strategies: selection of situations, modification of situations, deployment of attention, cognitive re-evaluation, and suppression of responses [33]. However, studies on emotional regulation in children with ASD during human–robot interactions are still lacking.

Since the appearance of the term EI, introduced by Goleman [29], the concept has received considerable interest from researchers. EI was formulated by Salovey and Mayer [34] and is made up of three components: the valuation and expression of emotion, the regulation of emotions, and the use of emotions. From the various definitions that have been provided for EI [29,34,35,36], three EI models have been proposed: (1) the ability model, (2) the mixed model, and (3) the trait model.

The ability model views emotions as useful sources of information that help make sense of and navigate the social environment. The model proposes that people vary in their ability to process emotional information and their ability to relate emotional processing to a wider cognition. Mayer and Salovey [37] proposed a model composed of four sets of emotion-processing mental abilities: (1) the perception, appraisal, and expression of emotion; (2) the emotional facilitation of thinking; (3) understanding and analyzing emotions; and (4) the reflective regulation of emotions.

The mixed model was constructed according to the definitions of Bar-On [35], with a more theoretical approach, whereas Goleman’s theory [38] is more practical. The mixed model by Bar-On is composed of five components: intrapersonal (self-regard, emotional self-awareness, assertiveness, self-actualization, and independence); interpersonal (empathy, social responsibility, and interpersonal relationships); adaptability (problem-solving, flexibility, and reality testing); stress management (stress tolerance and impulse control); and general mood (happiness and optimism). In contrast, the mixed model proposed by Goleman comprises five components: self-awareness (confidence and recognition of feelings); self-regulation (self-control, trustworthiness, and adaptability); motivation (drive, commitment, initiative, and optimism); empathy (understanding other feelings, diversity, and political awareness); social skills (leadership, conflict management, and communication skills).

Finally, the trait model was developed by Petrides [36] and is defined as a constellation of emotional self-perceptions located at the lower levels of personality. The model comprises four components: self-control, well-being, sociability, and emotionality.

### 2.4. Affective Interaction with Social Robots

Affective interaction with robots has received considerable attention in the field of human–robot interaction and affective computing. Affective computing, related to social robotics, enables robots to detect and understand human emotions and provide an intelligent and affective response. However, the detection of emotions in children with ASD is difficult because they suffer from a deficit in understanding the emotions of the other.

Hegel et al. [39] presented an anthropomorphic robot that perceives the emotional state of the user through speech and reflects the inferred state using a corresponding facial expression. An affective robot may use facial expressions and other non-verbal expressions to facilitate emotional communication, which involves expressing, perceiving, and understanding an emotional state. Therefore, for a robot to have the ability to express, understand, and perceive emotions, the development of cognitive-affective computational models is required. These models are also known as artificial agents that can perceive, understand, and express emotions.

However, some barriers limit the full capabilities of such models. One of them is that the robot cannot autonomously express its emotions according to its perceived environment, as different interaction situations may occur [40]. Therefore, it is common to find models that have been designed to respond in pre-determined ways to specific situations. It is challenging to integrate human characteristics and behaviors into a model because individuals have variations in their behavioral responses. Some of the models that have been proposed were inspired by theories of psychology, communication, social interaction, and artificial intelligence, amongst others.

The fuzzy logic adaptive model of emotions (FLAME) by El-Nasr et al. [41] is a computational model that uses a fuzzy-logic representation to map events and observations to emotional states. The model is based on the fact that the process of emotions can affect an individual’s decision-making, so it is composed of three components: an emotional component, a learning component, and a decision-making component. This model is based on the theories of Ortony et al. [27], Roseman et al. [42] (event-appraisal models), and Bolles and Fanselow [43] (an inhibition model). The authors used fuzzy logic to represent the intensity of emotions and to create a map of events and expectations of emotional states and behaviors. The FLAME was tested in a simulation of a pet named PETEEI [44].

Another model is the “fun empathetic agents reaching novel outcomes in teaching” (FearNot) by Aylett et al. [45], which is a computational model of emotions implemented virtually. It is centered on children for teaching about bullying and is based on the OCC theory [27] and that of Lazarus [24], with an appraisal theory of emotions approach. The authors tested their architecture only in controlled, bullying-focused situations. A layered model of affect (ALMA) [46] is a computational model of emotion that integrates concepts of emotions from the OCC theory and the three-dimensional model of emotion (arousal, valence, and dominance) from Meharabian [47], to generate states of mood and personality. It uses the Big Five model [48] and was implemented in a virtual 3D character. Another is the Emotion and Adaptation (EMA) [49], a computational model of emotions inspired by the appraisal theories of Smith et al. [50] and Lazarus [24].

The EMA is composed of five stages:

1. Knowledge representation, which is related to past, present, and future events as beliefs, desires, plans, and intentions.
2. Cognitive operators are related to computer metaphors, which can be cognitive, perceptual, or motor.
3. Appraisals consider appraisal theories, where each cognitive operator is represented using a casual interpretation considering that an event can be past, present, or future.
4. Emotions, mood, and focus of attention, which are appraisal patterns related to emotion labels
5. Coping strategies, which determine how the agent responds to the events.

However, the EMA has not been implemented and evaluated in a physical robot. Another model is the Empathetic PolarisX-based chatbot (EP-Bot) [51], which interacts through conversation. The EP-Bot is an empathetic chatbot that can better understand a person’s utterances. Conversation artificial intelligence technology is emerging in research in various fields, allowing communication through a dialogue based on emotions, identifying emotions from the utterance, and generating appropriate answers. Other chatbots exist, such as the Woebot, Wysa, Moodkit, and so forth, which are centered on mental health and where natural language processing is used following a cognitive behavioral therapy approach as a supplement to face-to-face therapy sessions to help reduce symptoms of depression and/or stress [52]. Chatbots are being used as emotional support in healthcare [53] and mental health [54]. However, in this type of interaction, emotions are usually expressed and recognized through the verbal/textual channel. There is only one interaction channel, but with a physical robot, different channels may exist through which to perceive and express an emotion.

None of these proposed models have been implemented in robotic systems, but only as virtual agents or conversational agents (chatbots or voice assistants). Some are only proposals and have not been assessed. We must remember that children with ASD prefer to interact with pictograms, which means that the current solutions are not optimal for them.

## 3. Objectives

The objective of this systematic review was to examine the literature of work that has been conducted on the intelligent affective interaction of social robots with children, especially intelligent interaction with children with ASD.

The objective was to provide a synthesis of the current research and to increase our understanding of the state of the art of computational models with emotional intelligence designed for physical robots used in interactions with children, especially children with special needs such as those with ASD.

The systematic review aimed to address the following research questions:

1. What is an intelligent method of affective communication for a social robot?
2. What theories/modules have been used to develop these models of affective communication?
3. Which of the proposed affective communication models have been used for children with ASD?
4. What are the differences between the affective communication models for children with ASD and those without ASD?
5. Can affective communication be achieved by SARs for children with ASD?

## 4. Methods

This review was conducted via a systematic search of the published literature available up to 2021, according to the guidelines of Preferred Reporting Items for Systematic Reviews and Meta-Analysis (PRISMA) [55].

Simultaneous searches were conducted in various research databases, including Scopus, Science Direct, Web of Sciences, and IEEE Xplore.

### 4.1. Review of Terms

A first search was conducted using “affective robot” and “autism” as search words in the Scopus and IEEE Xplore databases, up until 2021. Only four articles were found from 2014 to 2020, with only one published as a journal article and the other at conferences. The three conference articles do not propose any model of emotions; they analyze the affective responses of children with ASD when they interact with robots, but not autonomously. In 2020, Xiao et al. [56] designed an emotional interaction mechanism for children with ASD. They proposed a portable robot able to achieve deep emotional interactions with patients with ASD. This proposed portable affective robot perceives and expresses emotions. They also presented a multimodal data fusion method, which is one of the problems faced when data is captured from different sensors to perceive the emotion of a subject. The authors used visual, auditory, and physiological sensors (temperature and heart rate) to recognize the child’s emotion with ASD. To design the child–robot interaction, they used the emotional communication model proposed by [57]. However, Hirokawa et al. [58] mentions the problem with designing autonomous affective robots, since each child with ASD has different social and affective characteristics that must be considered. However, programming the robot’s behavior does not allow the therapist to personalize the activity according to everyone’s characteristics. Therefore, the question is, “How can one evaluate a study if the experimental protocol is different for each child?”

Finally, to further broaden the search, we used the terms “intelligent”, “emotions”, “robots”, and “children”. A total of 53 articles were found in Scopus, published between 1988 and 2020, of which 46 corresponded to conferences and 5 to articles; 1 was a book. In IEEE Xplore, 36 articles were found, of which 32 were conferences and 4 were articles published from 2005 to 2020. In Springer, 3287 were found, of which 892 were articles, 2316 corresponded to book chapters, and 717 to conference articles. However, only articles from the computer science and engineering disciplines were considered, for which a total of 573 articles published from 1987 to 2021 were selected. In the Web of Sciences database, 95 articles were found, from between 2003 and 2021.

We considered the terms in Table 1 for our search. The search for the terms was applied in article title, abstract, and keywords.

### 4.2. Inclusion/Exclusion Criteria Selection of Studies

The inclusion and exclusion criteria were determined prior to conducting the searches. The articles that were included in the review were (1) articles from disciplines related to computer science and robotics; (2) only articles, lectures, and book chapters; (3) models focused on physical robots (not an avatar computer or artificial agent). Excluded articles were (1) not available in English, (2) published in journals, or (3) unrelated to the purpose of the study.

## 5. Results

The initial search of the databases resulted in a total of 684 articles (53 Scopus, 36 IEEE Xplore, 573 Springer, and 22 WoS). Ultimately, only 46 articles published between 1997 and 2021 were selected, considering the inclusion and exclusion criteria. The selected articles allowed us to answer the study questions.

### Data Extraction

**Figure 1:**
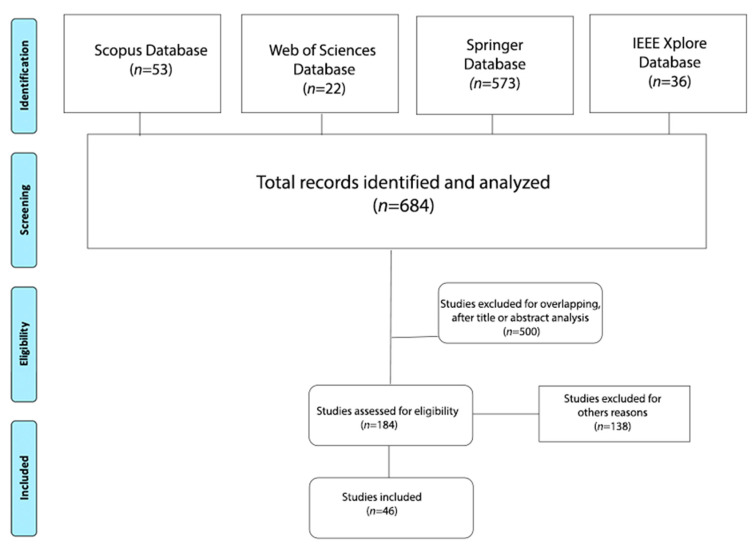
Flow of information of the systematic review process.

**Figure 2:**
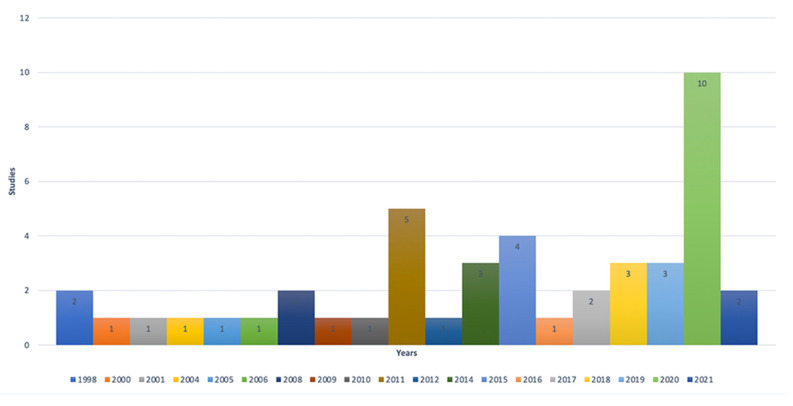
Number of studies analyzed in the review, grouped by year of publication.

Data were abstracted following the flow diagram presented in Figure 1, where 46 articles were selected, as described in Table 2. Figure 2 shows studies selected by year of publication, where studies of these affective models increased in 2020, which had 10 publications.

The objective of the abstraction was to respond to the following questions:

**Question** **1.** *What is an intelligent method of affective communication for a social robot?*

Human behavior can be influenced by emotions, which can internally affect some cognitive processes, such as perception, attention, and decision-making [28]. Externally, the emotional state of individuals is manifested by verbal and non-verbal communication, such as facial expressions, body posture, voice intonation, and physiological responses when interacting with others. However, there is no unique definition of affective communication. In the studies selected, we found several terms referring to emotion theories and computational models of robotic systems, such as artificial emotion [59], empathic robots [60], affective loop [61], artificial emotional intelligence, computational emotion, artificial empathy, and affective-cognitive models, and socio-affective architectures.

One field of study of emotional communication within computer sciences is affective computing (AC), which has become a more critical research term in human–robot interaction. However, studies have centered only on primitive interactions, such as facial expressions, body movements, or recognizing emotions through the robot’s sensors. However, affective communication is the robot’s ability to express, understand, and perceive emotions and, related to this, to make decisions about the environment. Thus, affective computing is when machines are developed to recognize, interpret, and process human experiences and emotions [62]. The first step in creating an affective machine is to use software and hardware with sensors that recognize emotions. The robot behavior is then adapted according to the recognized emotions of the person. Through behavior, emotions can be expressed, and decisions can be made according to the context, as shown in [63], where the authors state that empathic behavior has two levels of empathic responses: parallel and reactive. Parallel empathy describes empathic responses that mimic the target’s emotions, whereas reactive empathy describes empathic responses that foster objective verbal and non-verbal actions to reduce the target’s distress.

Different machine learning approaches are being used to build an empathy model, which involves recognizing and expressing emotions, as well as the ability to produce language, gestures, and postures to empathize with the subject. Many of the studies were centered on expressing emotions through facial and body expressions (non-verbal communication), but other functions have not been considered, such as interpreting, perceiving, and managing emotions, which are related to emotional communication. However, the term “intelligence” can be associated with emotional intelligence, as stated by Cominelli et al. [64], who proposed a socially intelligent robot capable of extracting meaningful information in real time from a social environment, so they constructed a system called Social Emotional Artificial Intelligence (SEAI) based on the emotional intelligence theory of consciousness of Damasio and the theory of somatic markers.

For communicating emotions, different channels must be considered to establish verbal and non-verbal communication, since the ability of a robot to change its voice, body pose, eye pose, and gestures to express its emotions and, in turn, respond according to the child’s emotions is more attractive compared to a robot that does not behave adaptively, according to Tielman et al. [65].

Velasquez [7] stated that computational models must be considered beyond their role in affective expressions. Several important issues should be considered, such as the differentiation of emotions from other affective phenomena with different durations in time, such as moods and personality; both have been associated with affect-congruent biases in emotional judgments. The emotional state can affect other systems and processes, such as attention, the bias of perception, and behavior.

In [66], the emotional communication of robots is related to some concepts, such as affect, personality, affective attitudes, moods, and emotions. “Affect” is an embodied reaction of pleasure or displeasure. Personality traits identify the consistent, coherent patterns of behavior and affects that characterize individuals. Affective attitudes are feelings about an object, a person, or an issue. Moods are low-activation states. Emotions are high-activation, short-term affective states and provide a fast, flexible response to the environment.

The study by [67] focused on the part of cognitive empathy by which empathy is specifically defined as the ability to understand and respond appropriately to the affective states of others. A socially assistive robot, thus, needs to (1) model the child’s affective states and (2) adapt its affective and prosocial behavior in response to the affective states of the child. However, several of the intelligent affective communications proposed do not focus on primary emotions. Such is the case for Cañamero’s [68] study, which focused on social robots in which artificial emotions were modeled, such as anger, boredom, fear, joy, interest, and sadness. Gadanho [69] related emotions to events, using the emotions of happiness, fear, sadness, and anger. Murphy et al. [70] presented artificial emotional states, including happy, confident, worried, and frustrated.

A study presented by [71] mentions that “emotional communication” between humans and robots must consider the following three factors to cause empathy:

- The robot does not need the standardization of the environment.
- The interface of the robot is not limited.
- The communication scenario is not set to the robot.

Hence, an autonomous robot was designed to express emotions considering metaphors of communication, such as speed, spatiality, and motion.

In summary, we confirmed that communication with robots is an exchange of information that can be verbal and non-verbal. This affective information is identified by the robot through sensors, and they respond to this information; when we mention intelligence, it is because the robot can adapt and learn behaviors in different environments.

**Question** **2.** *What theories and modules have been used to develop these models of affective communication?*

The selected studies indicate that there is a diversity of theories associated with emotions and social robots. We extracted a set of aspects to compare these models and theories based on some common aspects. Table 2 summarizes the comparison of the 46 studies selected, with descriptions of the set of aspects, such as the model name and modules considered in the model, theories used to support the model, emotions used in the model, outputs and inputs of the model, robot name, and if the model was designed for children with/without ASD.

Cathexis is a computational model of emotions [72] inspired by the theories of Damasio (emotional intelligence), artificial intelligence, and ethology. Initially, models were designed for virtual agents. Then, Cathexis was adapted for a physical robot called Yuppy, for which a set of needs was defined, including recharging, temperature, fatigue, and curiosity, representing senses from different sensors. The Cathexis model is composed of four principal modules: the emotion generation, behavior, drive, and motor systems. The emotion generation system module was influenced by Izard’s multi-system for emotion activation [73]. Emotions are expressed using facial expressions, which create six different emotions: anger, fear, distress/sadness, enjoyment/happiness, disgust, and surprise. For the expression of emotion, Ekman considered universal facial expression [17]. Other models based on Damasio’s theory on somatic markers are described in [64,74,75]. The models proposed include drives and emotions and emotional memory.

The emotion generation system module in Cathexis [72] has a set of releasers that constantly check the right conditions to trigger the emotion they belong to. The releasers are neural, sensorimotor, motivational, and cognitive. Each emotional system includes two thresholds, such as (1) α, used to determine when an emotion occurs, and (2) ω, which specifies the level of saturation for that emotion. Meanwhile, the behavior system is related to reasoning and decision-making, through which an agent must choose how to respond to a situation according to the environment. In addition, Cathexis considered moods and temperaments based on concepts proposed by Minsky [76]. Meanwhile, [75] also considered other theories as ethological and psychological models of behavior. Hence, the model is composed of an emotional system based on somatic markers and a cognitive system responsible for the perception of a robot, object tracking, memory, attention, behavior, and motor coordination. The robots have different appearances; one is anthropomorphic, while the other is not. The latter expresses emotions with other metaphors of non-traditional communication, for example, fatigue is related to the battery level. In both models, the details of how emotional memory should be mapped were not sufficiently provided.

The model EMOBOT [74] was inspired by theories of control and Damasio. This model only has one module called the controller, which internally has internal values and action selection. The EMOBOT has three levels of control. The high-level control behaviors for autonomous robots are tasks with linguistic commands, such as “deliver the email”, “go and count the number of chairs”, and so forth. The internal values of the controller are inspired by the theory of Damasio and are related to driving values, such as fatigue, hunger, homesickness, and curiosity, which are defined as primary states. Meanwhile, emotions are considered to be secondary states, such as fear, anger, boredom, and happiness. The representation of knowledge is inspired by theories’ fuzzy control, rules, and differential equations. Hence, the mapping of emotional memory (action selection) is given by a multidimensional matrix quantized into four regions: very low (−1.0, −0.5), low (−0.5, 0.0), high (0.0, +0.5), and very high (+0.5, +1.0).

Another model is SEAI (Social Emotional Artificial Intelligence). Cominelli et al. [64] were inspired by the theory of consciousness of Damasio, which describes emotion as a neural reaction to a certain stimulus, realized by a complex ensemble of neural activations in the brain. In other words, inputs from sensors are considered the knowledge structures that allow reasoning. These inputs can determine reactions, and the actions can be the internal or external determination of the reasoning process. SEAI is composed of three main functionalities: SENSE, PLAN, and ACT. The ACT is the robot actuation system, with functionalities such as the configuration of servo motors to express emotions through the face and body. The somatic marker is integrated into the PLAN block, which corresponds to a set of rules working in two directions: analyzing the body and emotional state to trigger the assertion of the somatic marker. In case of recognition of a marked entity, they can recall the bodily state that the agent felt when that entity was labeled. However, the authors do not describe how the emotional state is modeled. SEAI was embodied in a humanoid robot called Abel [77]. The authors used Russell’s circumplex model with two coordinates (valence and arousal), which is useful but limited, because it does not allow for the expression of higher levels of emotional states, such as the mood of the robot.

A biological model of emotional communication is embodied in the WAMOEBA robot [71]. This robot can recognize emotions through voice and facial recognition. This model is based on the endocrine system, which has the function of creating homeostasis. Homeostasis evokes an internal body state, such as tension in the muscles, shrinking of the pupils, temperature, and so forth. Some examples of homeostatic feelings are thirst, hunger, desire, please, and well-being, amongst others. Humans can communicate with WAMOEBA with various reactions, such as approaching, escaping, making sounds, eye tracking, and arm stretching. WAMOEBA is a robotic arm, designed to express emotions through changes in the speed of movement, the volume, speed, and loudness of sounds, and the color output on an LCD using hormone parameters. WAMOEBA can detect four emotions: anger, sadness, pleasure, and expectation. Its characteristics of communication are (1) adaptability to the environment, (2) diversity in the ways to communicate, and (3) development of communication according to the behavior of humans. Other models found that are based on the hormone system are described in [69,78,79]. The hormone system [69] is based on Cañamero’s proposal [68]. The models [69,71] are embodied on robots, not humanoids, and express affective communication through metaphors of non-verbal communication, such as movement, speech, temperature, battery level, colors, and orientation, among others. However, the emotional model of [69] considers feelings and sensations. In addition, it can detect four emotions: happiness, sadness, fear, and anger. However, the emotional state can be influenced by the robot’s feelings, such as hunger, pain, restlessness, temperature, eating, smell, warmth, and proximity. Each hormone is associated with each feeling, while in the model of [71], each hormone is associated with an emotional state. Meanwhile, the model of [79] has a homeostatic regulator, following the animal approach. The homeostatic regulator simulates physiological variables as hydration or glucose levels. The robot reacts to the physiological state perception. Each physiological variable has levels, for example, food gets a low level, so the hunger drive gets high. We can also find mixtures of biological and psychological. The endocrine system of [78] consists of two layers: emotional and biological hormones. Biological hormones are represented as blood glucose, body temperature, and appetite. Emotional layers are six emotions, including happiness, sadness, disgust, surprise, anger, and fear, and six moods, including sleepy, tired, embarrassed, hungry, bored, and loving. The Lovotics robot was designed and developed using theories of hardware and software. The model is composed of three modules:

- Perception, which captures sensory data, including sound, vision, touch, and acceleration.
- A processor, which functions to analyze data and apply techniques of artificial intelligence, amongst others.
- Outputs through various channels: vision, audio, color, and motion.

The artificial intelligence of robots is used for the formulation of love. An artificial endocrine is implemented in the robot to imitate human endocrine functionalities. Additionally, the system has a probabilistic love assembly and affective state transition modules. For calculating the love between humans and robots, some parameters are considered, such as proximity, repeated exposure similarity, desirability, and attachment reciprocal linking, among others.

Another study inspired in biological systems is the one done by [59]. The artificial emotional model is based on the hierarchical structure of the human brain. The author defines a hierarchical model based on former emotional experience. In addition, it is derived from unconscious judgments. The subject finds a new event during its learning from the environment and acquires a positive or negative emotional experience, and the new event can be added into a series that activates emotion. According to the definition of the model proposed by [59], it is very similar to Damasio’s theory of “Somatic Markers”; this is because they considered a neuroscience approach. This model is based on a child playmate robot. The model uses neural network reinforcement as a learning mechanism, using positive reinforcement (positive emotional experience) and negative reinforcement (negative emotional experience).

Reinforcement learning is a way to represent the learning in social robots, which is a framework for decision-making problems, in which the learning robot senses the current state and chooses an appropriate action. The environment changes its state to the succeeding state according to the probability function. We also can find studies using a reinforcement learning algorithm, such as those by [79,80,81,82]. The study by Bagheri et al. [81] was based on the cognitive empathy framework for social robots. Their model can express, perceive, and understand emotions. The model is based on the cognitive-effective constructs of Davis [83], which explain the processes and outcomes of empathy. The framework contains three modules:

- Emotion detection, which detects and recognizes an emotion from facial expressions.
- Reinforcement learning algorithms, through which, over time, they learn to select the empathic behaviors that comfort users in different emotional states.
- Empathic behavior provider, which applies selected behaviors to the robot to react to users’ emotions.

In 2015, Johal et al. [84] proposed the Cognitive and Affective Interaction-Oriented (CAIO) architecture for SARs. The architecture was inspired by the Belief, Desire, Intention (BDI) model [85]. This model is based on Bratman philosophical theory [86], which explains reasoning through attitudes such as beliefs, desires, and intentions. Beliefs represent characteristics that are updated after the perception of each action. Desires represent the motivational state of the system, related to the goals to be achieved. Intentions represent the current action plan chosen. CAIO has two loops: deliberative and reflexive. The deliberative loop is used to reason, has five mental states called Beliefs, Ideals, Goals, Responsibilities, and Emotions (BIGRE), and produces plans of action. The reflexive loop is responsible for emotional reactions. CAIO has five modules: multimodal perception (visual and audio sensors), memory, appraisal, deliberation, planning, and multi-modal action renderer (physical). CAIO was developed for children that can interact with a companion robot, which was embodied in a Nao Robot. The BDI model inspired CAMAL [87]. CAMAL is embedded into a mobile robot, where reactions or expressions of the robot vary according to deliberative goals or environment. The model contains a module called “BDI schema” that is implemented using associations. Each association is composed of a belief–desire–intention triplet, with the following form: association (found(ball), hit(ball) moveTowards(ball), 0.25), where the value details the likelihood that the intention of a given association will achieve a goal, given a specific belief.

To achieve smart social interaction, robots need the ability to recognize and express emotions, which can be verbal and/or non-verbal signals. Hirth and Berns [88] designed an emotion-based architecture for social robots, investigating how social interactions between humans occur. The authors were inspired by the theories of emotion and motivation [89]. Behavior-based Control (iB2C) [90] was designed for robot behavior. The architecture was tested on the humanoid robot ROMAN, which is equipped with 24 degrees of freedom (DoF) to express emotions through non-verbal signals. This model includes four modules: percepts of interaction, habits of interaction, motives, and emotional state, which is represented by the three dimensions, arousal (A), valence (V), and stance (S), and which is very similar to PAD model [46]. The interaction habits describe the expression mechanism of the robot (eyebrow up, mouth corner back, etc.), while the perception captures the environment through different sensors applying the technology of multimodal fusion. Each module is a vector with three inputs—stimulation, inhibition, and data input—and three outputs, including activity, target rating, and data output.

iGrace [91] is an extension of the GRACE (Generic Robotic Architecture to Create Emotions) model [92], and is a computational model of emotion-focused emotional expressiveness and personality. The robot reacts according to the speech of the speaker. The GRACE model was inspired by the theories of the psychology of Ortony, Clore, and Collins (OCC) [27], which is an appraisal approach. OCC selected 22 emotional states according to the situation type. This model is based on valuation theory, where there are sources of different value types, such as goals, standards, and tastes; each one has a different domain, such as events (e.g., joy and pity), actions (e.g., pride and reproach), or objects (e.g., love and hate). The three domains are related to affective reactions, such as being pleased or displeased at the outcomes of events, approving, or disapproving actions, and liking or disliking the attributes of objects. GRACE is supported by the theories of Lazarus [24], Scherer [93], and Myers–Brigg [94] on personality. This model is composed of three parts: input (sensors), emotional interaction, and expression of emotions. The emotional interaction module is composed of four parts:

1. The moderator represents the cognitive internal emotional state. It builds a list of emotional experiences as a personality and mood.
2. The emotional experiences selector represents the emotional state. It builds a list of emotional experiences and functions from the words of the discourse.
3. The emotional experiences generator represents cognitive internal emotion.
4. The behavior chooses a reaction according to the best emotional experiences.

To express emotions, the six primary emotions described by Ekman were considered. The expression of emotions is given by a matrix between emotions and emotional experiences. The emotional expression of the robot is realized by the actions of buzzers and motors. Motors are related to movements to express facial expressions, including joy, surprise, sadness, anger, fear, and disgust. The selection of facial features is considering the EMFACS system.

Another key aspect is the processing of verbal and non-verbal information. This study proposed a multimodal affective computing approach for children [95] and incorporated this aspect using the RULER theory [96] to regulate emotions. RULER follows an approach to Social and Emotional Learning (SEL), which promotes the development of five key emotion skills: recognizing, understanding, labeling, expressing, and regulating emotions. The interaction of the robots includes visual and verbal information. The model has the following modules: multimodal data capture, data pre-processing, affective computing, cognitive computation, and output.

The affective loop is the affective model for social robotics, where the robot can adapt its behavior according to the needs and preferences of the user. The design was inspired by the theories of emotional intelligence described by Hoffman [97] and Goleman [29]. This model is composed of three modules: perception, management of emotions, and expression. Another model based on the theory of Hoffman is that by [67], which is composed of three modules: affect detection, empathic appraisal, and action selection. It was applied to the iCat robot to interact with children. It has two databases: supportive behaviors and memory of past interactions. The supportive behaviors are based on the theory of Cutrona et al. [98]. Meanwhile, the loop affective model has a visual system that monitors the user’s interest in the interaction. For example, if it detects that a child starts to be bored during a scenario, the robot stops the activity and entertains the child (e.g., dancing).

To achieve more extended interaction, the Automatic Cognitive Empathy Model (ACEM) was proposed for humanoid robots. Bagheri et al. [63] considered the definition of empathy proposed by Davis as “a set of constructs that connects the responses of one individual to the experience of another” [83]. They considered two kinds of empathy: cognitive and affective. Considered affective factors of empathy were gender, personality, age, and past experiences of the empathizer, which can affect the type of empathy they express. ACEM is composed of three modules: (1) emotion detection, (2) perspective-taking, and (3) empathic behavior provider. To recognize emotions, facial recognition techniques were used, and emotions were expressed through body motion speed and eye-color change, light green, blue, and red for happiness, sadness, and anger, respectively, and orange, purple, and dark green for surprise, fear, and disgust, respectively. The authors mapped the facial features of the robot according to the emotional state. The detection module was built with a deep neural network. To enable the robot with emotional energy, the robot’s considered parameters were speech (rate, volume, and pitch), body (motion and speed), and eye color (duration and intensity). The range of each value is considered according to the personality of the robot (introvert or extrovert). It was tested in the Pepper robot.

iCub is a humanoid robot [99], which has a cognitive-affective architecture. The architecture is a kind of loop, which is composed of (1) perceiving the emotional state, (2) predicting which action would be the most beneficial for the robot and human, and then performing the most beneficial action, and (3) evaluating from the perceptual input if the person’s reaction was predicted, modifying the belief values if wrong and reinforcing them if right. iCub looks like a small child and has the physical and cognitive abilities of a child. In other studies, iCub is beginning to be used in children with autism to learn motor communication through imitation [100].

A statistical approach based on HMM (hidden Markov model) was found in the study by Liu et al. [32]. The authors implemented the emotional interaction with facial expressions and behaviors (head and arms) in the robot. The aim of the model is the emotional regulation based on the Gross cognitive process [31]. Gross proposed five emotional regulation strategies, including situation selection, situation modification, attention deployment, cognitive reappraisal, and response suppression. Thus, the robot has an initial emotional state with calming. The robot perceives an external stimulus as “disgust” and compares it with its own current emotional state and then the output of the emotional state has 26 possibilities. Each emotional state corresponds to a point in the emotional state space associated with three parameters as direction vector, coefficient, and intensity of the emotional source. Meanwhile, the EMIA (Emotion Model for Intelligent Agent) [101] based on the control of complex systems uses fuzzy logic to handle uncertain and subjective information. In addition, the model was inspired by the appraisal theories of emotions, the emotion regulation theory (Gross theory), and multistore human memory. The appraisal variables are defined according to three theories: OCC theory [27], Roseman theory [26], and Scherer theory [93]. The model categorizes emotions into three groups, comprising consequences of events, actions of agents, and aspects of objects. Moreover, several studies work the emotion as a discrete model. However, this model considers the emotion as a continuous entity, as iGRACE does [32,91,92]. Some models have considered past experiences of an event/object. However, the EMIA has designed three types of memory for various processing and learning tasks, including perceptual memory, working memory, and long-term memory. The emotion modeling was created using fuzzy logic due to emotions being very complex and uncertain. The model was designed but it has not been evaluated in a robotic system.

Other approaches have been proposed to integrate emotions with cognitive architecture. Pérez et al. [102] developed a cognitive-affective architecture for ECAs (Embodied Conversational Agents), which was inspired by the ALMA [46] and Soar [103] cognitive architectures. The model is based on emotions, mood, and personality, which present short, medium, and long-term affective characteristics. Emotions are mapped onto PAD values. However, the architecture is oriented on conversational agents and not robotic systems. Additionally, in 2017, Tanevska et al. [99] proposed an affective cognitive architecture for the iCub robot. iCub can perceive and evaluate emotional states. The process of the model is (1) perceiving the state of the subject, (2) predicting which action is most beneficial for the robot and human, and (3) evaluating the perceptual input reactions of the subject and modifying the belief values. The authors considered the functionalities necessary for cognition were learning and intelligence. Thus, they implemented a memory module using reinforcement learning algorithms. The architecture was tested to detect and track the affective state of the users.

A technique for the communication of social robots called ERIK (Expressive Robotics Inverse Kinematics) was proposed by Ribeiro and Paiva [104], whose objective was emotional expression. The model is focused on the emotional expressiveness of an object, such as an arm. The expressiveness of the robot is related to expressive kinematics, that is, angles for each degree-of-freedom to represent a posture movement, which was tested on the Adelino robot, a robotic arm. However, the movements’ expressivity with affective states was not explored in depth in the study. Following this approach on communication metaphors, a model was proposed to express artificial emotions using color, motion, and sound. Löffler et al. [105] were inspired by the cognitive-linguistics theory of conceptual metaphor and emotion proposed by [106], which can be captured through the analysis of metaphors in discourse. For example, the emotional state of joy is warm, and temperature can be used to express emotions in robots.

The START framework was designed through a more therapeutic approach [107]. START is embodied in a Moxie robot for children with ASD and it helps promote social, emotional, and cognitive development through play-based learning. However, Moxie’s therapeutic framework is based on situation, task, action, result (STAR). Since it aims to help improve social and emotional skills in children with ASD, the framework is more focused on a therapeutic approach based on cognitive behavioral therapy (CBT) and naturalistic applied behavior analysis (nABA). Moxie was designed to help children, especially those with ASD, to learn and safely practice essential life skills, such as turn-taking, eye contact, active listening, emotion regulation, empathy, relationship management, and problem-solving. To express emotions, the creators designed an expressive face for Moxie, with large and friendly eyes as a stand-out feature. Likewise, the ears were designed to visually signify that Moxie can hear so that the children can whisper into its ear. However, the description concerning the structure of Moxie’s architecture is vague. Following this approach, the First-ECS (Emotion Care System) for emotional communication [56] was proposed for children with ASD. The aim was to improve the emotional perception and expression ability. Understanding the emotion is considered data from different channels, including the auditory, physiological (respiration, EEG, temperature, heartbeat, and respiration), and visual. The data provided by multiple sources, also known as multidimensional data, are applied machine learning techniques to generate high-quality emotional information. However, few models provide information about how they can affect the data combination to recognize emotions.

Social robots need to be able to interpret human affective cues. However, an emotional state can be recognized through several cues, such as auditory, visual, or physiological. Robots can use one or more sensors (camera, microphone, pressure, and physiological) to recognize an emotion. Some models are considered multimodal emotional, such as the model proposed by [108], a novel multimodal emotional architecture designed to promote natural and engaging bidirectional emotional communication between social robots and humans. Emotional communication is detected using a combination of modalities such as body language and vocal intonation. To express emotions, the robots use communication modalities such as eye color, body language, and speech. Following this approach, Aly et al. [109] designed an expressive ALICE robot that generates an adapted multimodal behavior to enhance the interaction with a human. The study was focused on emotional expressivity in terms of body gestures, speech, and facial expressions. For facial expressivity, a coding system of facial actions (FACS) was considered [110].

Other studies have integrated personality, such as TAME [66,111,112], iGrace [91], EMIA [101], and ECAs [63,102]. These models have used the Big Five model [48] and OCEAN, representing the following five dimensions: openness, conscientiousness, extraversion, agreeableness, neuroticism. The authors of [63] incorporated two types of personality into the robot, extrovert and introvert.

In the selected studies, the models of psychology most often used to design the computational models were Scherer [113] and Smith and Lazarus [50]. Scherer considered emotions as a multicomponent process, of which the cognitive component is one, introducing an appraisal process as a sequence of stimulus-processing steps. Smith and Lazarus [50] proposed a model based on cognitive–motivational–emotive theory. Appraisal theories state that emotion is related to two basic processes: appraisal and coping. Appraisal is the process through which a subject can evaluate the relationship with its environment and can be affected by past events. The appraisal outcomes can be tendencies, subjective experiences, or physiological responses, such as facial expression, posture, and so forth. Coping activities are related to the action tendency, which can be related to personality.

However, from a neurobiological point of view, several researchers are integrating emotional intelligence based on Damasio’s theory about the somatic marker hypothesis. The somatic marker is associated with decision-making theory, in which an emotion can be associated with past experiences. Damasio defined the somatic marker as “the somatic marker forces attention on the negative outcome to which a given action may lead, and functions as an automated alarm signal. The signal may lead you to reject immediately the negative course of action and thus make you choose among other alternatives” [28].

The theory shows how emotions play an essential role in decision-making. Damasio described this course of events with five steps:

1. An emotion can be induced by several sensorial channels: visual, auditory, and tactile, amongst others.
2. Signal processing of the different sensory channels can activate neural sites that are present to respond to the particular channel.
3. An emotion can be manifested in different psycho-physiological responses.
4. Changes in body state are represented by both the subcortical and cortical regions, which are represented by first-order neural maps.
5. An emotional state neutral is represented by second-order neural structures.

**Question** **3.** *Which of the proposed affective communication models have been used in children with ASD?*

We found three studies of affective communication models for children with ASD. These studies have different approaches. Kozima et al. [60] proposed a robotogenetic model inspired by the theory of mind and an ontogenetic approach. Xiao et al. [56] focused on helping them to improve their emotional interaction ability through audio and video perceptions. This study explored artificial intelligence (AI)-based algorithms, fusion methods of multimodal data, and relationships between multimodal data and emotions. The emotional communication of children with ASD is unequal to those of the communicators. The authors explored different data, including video, audio and physiological. However, in the physiological cues, they do not describe which cues are most relevant for recognizing an emotion in children with ASD.

A study conducted by Cohen et al. [114] mentions that children with autism have major difficulties in recognizing and responding to emotions and mental states in others’ facial expressions. This indicates that affective communication for children with ASD involves not only designing affective computational models but must also be subject to the physical appearance of the social robot. The Moxie robot and [107] and the Abel robot [77] were considered for the expressiveness of emotions. Abel is a humanoid adolescent robot that was initially designed to investigate social interaction and human cognition. This robot was equipped with sensors and actuators to detect and express emotions at a high level of realism, and has inspired facial expressiveness that has been used in therapy for children with ASD [115]. Abel has a cognitive system based on SEAI, inspired by Damasio’s theory of mind and consciousness. In addition, the Moxie robot is based on computer vision concepts to express emotions through a representation of a 3D face with a screen. However, Moxie is not only designed to help promote emotion but it also includes social and cognitive development, using play-based learning as a strategy for interaction. Moxie was based on theories of therapies for children with ASD, including cognitive behavioral therapy (CBT) and naturalistic applied behavior analysis (nABA), which are used for social and emotional skills training.

Affective communication for children with ASD is related to a cognitive component, which consists of the recognition of another person’s mental state. This is known as the theory of mind. Kozima et al. [60] designed two robots, Infanoid and Keepon, in which the functions of eye contact and joint attention were implemented. Both functions are used to develop the capability of empathetic communication through physical and social interaction. Infanoid is an upper torso humanoid robot, composed of 29 actuators and several sensors, with most of their movements are centered on facial expressions. Keepon is a small non-anthropomorphic robot that expresses its attention by orienting its face and exhibits its emotional states through its body from left to right. Both robots have cameras to evaluate eye contact capability in real time. If a face is detected, the robots drive to direct the gaze/face/body toward the detected face. Joint attention was also implemented, in which the robots first generate several hypotheses of the direction of the face being tracked. From images taken by the cameras, the likelihood of each of the hypotheses is calculated and the best direction is selected.

**Question** **4.** *What are the differences between the affective communication models for children with ASD and those without ASD?*

We found ten studies focused on children without ASD. These studies cover different proposals, including learning [61,116], emotional regulation [32], shared attention [75], playmate [59,67,117], and companion [84,91,95]. The model by [79] was inspired by emotional communication for infants, as studied by Feinman et al. [118] (social interaction) and Davies and Stone [119] (shared attention). The robot is able to interact and communicate through speech, gestures, and facial expressions. Truschzinski and Mïller [79] were also inspired by the computational model of the Kismet robot [120], the first social, emotional robot. The Leonardo robot uses a simulation theory that infants learn to decode emotional messages conveyed through facial expressions by leveraging their early facial imitation capability to bootstrap emotional empathy. It is supported by Meltzoff [121], who affirms that infants have the ability to imitate facial expressions, thus, the Leonardo robot can imitate the facial expressions of others. Children with ASD have problems related to the perception, understanding, and expression of emotions. Therefore, the appearance of a robot must be considered as an aspect essential for emotional communication. However, studies designing robots for children with ASD are still unclear on the ideal appearance of a robot. In addition, social interaction and shared attention strategies are different for children with ASD. They have many difficulties with shared attention, and hence, joint attention [122] therapy focuses on improving specific skills related to shared attention, such as coordinating looks between a person and an object, pointing, and playing games, among others.

Another study by [32] was based on emotional regulation for typical children. The authors were inspired by the Gross theory and the Weber–Fechner law. However, the authors did not consider the child’s cognitive development and social constructivism. This is because the study focused on micro-expression cognition and emotional regulation based on the Gross theory. Moreover, this model was applied to universal psychology without taking into account emotional changes. Comparing atypical children, there are differences in emotional regulation (ER) expression. They have different ER strategies and rely more on others to regulate their emotions than their typically developing peers. In addition, ASD symptom severity and low executive functioning are associated with poorer ER abilities [123]. Thus, these same strategies used by [32] cannot be used for children with ASD.

We also found several studies in which these models (iGrace, affective loop, and CAIO) were designed as companion robots for typical children. They incorporated personality and moods into the robots. However, these studies do not describe whether they can support long-term interactions with children. Only one study was found [67]. The study was inspired by Scherer’s theory [93] and the theory of supportive behaviors [96], which includes actions to reduce others’ distress. For example, the iCat robot has a set of supportive behaviors that it can employ when the child’s affective state is negative. The evaluation showed that children perceived the robot as more engaging and helpful when it reacted to their emotions. The companion robots for atypical children are being designed to diagnose autism [124]. It is also important to consider that a robot’s personality and moods can make it more autonomous in its interactions. They could cause greater curiosity in children with ASD and could serve as support tools for assistive therapy sessions.

**Question** **5.** *Can affective communication be achieved for SARs for children with ASD?*

Studies showed that the clinical use of robots can provide an alternative for children with ASD [38,107] (1) to understand behaviors [99], (2) to understand the emotions [107], and (3) to regulate emotions [38], amongst others. SARs can help to provide feedback on performance.

In 2019, Cañamero [125] mentioned that computational models of emotions can provide the possibility to develop, test, extract, and analyze models and emotional theories. However, designing and implementing these models is challenging, involving different areas, such as electronic sciences, computer sciences, and theories of psychology, amongst others. In turn, the design of these models is subject to the behaviors we want to give the robot to express itself or communicate non-verbally.

Autonomous robots with embodied emotional models have more natural interactions, creating a level of trust between the robot and humans. Therefore, they may have the potential to influence how children develop empathy, and even more so for children with ASD, who have social interaction deficits. However, the conducted studies are experimental and controlled, and the impact of these SARs on children with ASD is still uncertain. Leite et al. [67] explored the role of empathy in long-term interactions between children and social robots. They argue that artificial companions capable of behaving in an empathic manner would be more successful in establishing and maintaining a positive relationship with users in the long term. That is, a social robot can help children with ASD with the development of social skills. Paiva et al. [9] presented that the ability of robots to interact with humans in ways that resemble human interactions is becoming increasingly more relevant. Emotions are essential for that interaction but computational models are required to express and recognize emotions. The authors defined an affective loop with an interactive process. The user first expresses their emotions through a physical interaction involving their body, and the system then responds by generating affective expressions, such as colors and haptics, among others.

However, other aspects that can influence the acceptance and usage of the social robot are its appearance and communication method. The selected studies used verbal communication through visual channels, such as facial expressions and body movements (head and hands). They also used other forms of expression, as in the case of Yuppy, where a set of needs was defined: recharging, temperature, fatigue (battery of robot), and curiosity, representing senses from different sensors of the robot. Other researchers [126] proposed a multimodal expression of emotion using color, motion, and sound.

The selected studies show that social robots have different shapes or functions, but they must recognize the presence of a child to engage in social interactions, express their own emotions, and understand the interactions. However, further studies are required.

**Table 2:** Summary of published research on affective communication models in social robots.

Year	Title	Model/Architecture (Modules/Name)	Theories Inspired	Emotions	Outputs/Inputs	Robot	Child	Child with ASD
1998 [72]	Modelling emotions and other motivations in synthetic agents.	Cathexis: - Emotion generation - Behavior system - Drive system - Motor system	Damasio’s theory: decision-making; ethology theories; artificial intelligence theories.	Anger, fear, distress/sadness, enjoyment/happiness, disgust, and surprise.	Battery, temperature, energy, interest levels Cameras, Audio, IR sensors for obstacle, air pressure sensor	Yuppy	No	No
1998 [127]	Intelligent agent system for human–robot interaction through artificial emotion	- Rational - Emotional - Reactive	Multimodal environment, model of artificial emotion using Kohonen’s Self-Organization Map (SOM).	Tranquil, happy, melancholy, angry.	Movement, light and acoustic (music and sound) Camera, ultrasound sensor	Pioneer 1 Mobile Robot	No	No
2000 [71]	Emotional communication robot: WAMOEBA	Endocrine system: - Four hormone parameters - (H1, H2, H3, H4).	Behavior of robots could be interpreted as feelings, based on the Urge theory of emotion and cognition proposed by Toda [128], model of endocrine system of humans.	Anger, sadness, pleasure, expectation.	Actuator speed, LCD color, cooling fan Camera, sound (volume, speed, pitch)	WAMOEBA- 2R	No	No
2001 [129]	Robot learning driven by emotions	- Hormone system - Dominant emotion - Emotions - Feelings - Sensations.	Perception, reinforcement, and control triggering. Emotions influence the feelings through a hormone system.	Emotions: happiness, sadness, fear, and anger. Feelings: hunger, pain, restlessness, temperature, eating, smell, warmth, proximity.	Battery, light, motor speed Proximity sensor	-	No	No
2001 [130]	Model of knowledge, emotion, and intention	K.E.I: - Knowledge - Emotion - Intention	Algorithm Q-learning to learn a series of behavior patterns. Fuzzy Cognitive Maps [131]	Anger, fear, abandonment, avoidance, troublesome, anxiety, approach-forward.	Camera	-	No	No
2004 [60]	Can a robot empathize with people?	Robotogenetic: - Joint attention mirror system - Project of mental states - Estimation of mental states	Theory of mind, development of empathy of the child.	Reading of desired or negative emotions of the infant.	29 actuators (face and body), speech synthesizer Cameras and microphones	Infanoid, Keepon	Yes	Yes
2005 [75]	An embodied computational model of social referencing	- Attention - Belief system and affective appraisal - Emotion system - Action system Shared attention mechanism: robot attentional focus; human attentional focus; referential focus	Theory of Damasio, theory of OCC, dimensional theory (arousal, valence), and human infants.	Happiness, surprise, contempt, sadness, fear, disgust, and anger.	65 actuators, facial and body expressions Camera, microphone (vocal intonation)	Leonardo	Yes	No
2006 [74]	EMOBOT: A Robot Control Architecture Based on Emotion-Like Internal Values	EMOBOT: - Controller (internal value system, action selection) - Levels of control: low–mid (1–2) and high.	Neuronal network and neuronal learning paradigms. Theory of control, theory of psychology of Damasio.	Primary internal states (drives): fatigue, hunger, homesickness, and curiosity. Secondary internal states (emotions): fear, anger, boredom, and happiness.	Movement directions (motors) ultrasonic sensor, ambient light, infrared	-	No	No
2008 [117]	An affective model applied in playmate robots for children	- Behavior database - Distributed cognitive information processing - Personalization interaction - Multimodal characteristics fusion - Recognizing feature extraction	Based on HMM.	Happiness, anger, and sadness	-	-	Yes	No
2008 [132]	Multi-dimensional emotional engine with personality using intelligent service robot for children	- Reactive dynamics - Internal dynamics - Emotional dynamics - Behavior dynamics - Personality	Dimensional theory of emotions and personality model using five factor models in psychology [48].	Happy, sad, surprise, disgust, fear, angry.	Temperature, speech, facial expression, humidity Camera	iRobi-Q	Yes	No
2009 [88]	Emotion-Based Architecture for Social Interactive Robots	- Motives - Emotional state - Habits of interaction - Perceptions of interaction	Theory of social interaction Watzlawick [133], theory of motivation [89], iB2C architecture [90].	Anger, disgust, fear, happiness, sadness, surprise. Motivation such as: obeying humans, self-protection, energy consumption, avoid fatigue, communication, exploration, and entertainment.	Facial expressions, head (up/down) Camera, microphones	ROMAN	No	No
2010 [87]	Robo-CAMAL: a BDI, motivational robot.	CAMAL: - Affect model - Motivation blackboard - Motivator update - Reasoning module - BDI schema	Psychological (belief–desire–intention) BDI model, CRIBB model (children’s reasoning about intentions, beliefs, and behavior) [134].	Drives, goals, desire, intentions, and attitudes.	Movement directions Camera, microphone	Mobile robot	No	No
2011 [91]	Children recognize emotions of EmI companion robot	iGrace: - Inputs - Emotional interaction - Expression of emotions	Based on the GRACE model. The EMFACS system is used for the facial expression of emotions.	Joy, surprise, sadness, anger, fear, disgust.	Facial expression (mouth, eyebrows, ears, eyes), tone of voice, posture (movement, speed) Camera, microphone	EmI	Yes	No
2011 [59]	Artificial emotion model based on reinforcement learning mechanism of neural network	Homeostasis and extrinsic motivation, appraisal, and intrinsic motivation. Reward and value function and hard-wired connections from sensations.	Reinforcement learning and based on the hierarchical structure of human brain.	Emotional polarity	-	-	Yes	No
2011 [66]	TAME: Time-Varying Affective Response for Humanoid Robots	- Dispositions - Affective state - Active behavior - Behavior coordination - Perceptual module	Personality, emotion, mood, and attitude areas of psychology.	Fear, anger, disgust, sadness, joy and interest. Personality: openness, conscientiousness, extraversion, agreeableness, and neuroticism.	Facial expressions Body expression (head, ears, movement), LED	AIBO, NAO	No	No
2011 [135]	A layered model of artificial emotion merging with attitude	AME (Attitude Mood Emotion): - Attitude layer - Mood layer - Emotion layer	OCC theory, PAD (Pleasure Arousal Dominance) emotion space.	Happiness, dependence, calm, mildness.	-	FuNiu	No	No
2011 [136]	Emotions as a dynamical system: the interplay between the meta-control and communication function of emotions	- Drives, self-monitoring, and emotions - Self-monitoring and meta control	Canon–Bard theory, model of emotions of FACS (Facial Action Coding System).	Interest, excitation, satisfaction, joy, hunger, fear, shame, and disgust.	Movements, camera	Mobile robot	No	No
2012 [78]	A Multidisciplinary Artificial Intelligence Model of an Affective Robot	- Probabilistic love assembly - Artificial endocrine system. - Affective state transition	Dynamic Bayesian network.	Happiness, sadness, disgust, surprise, anger, and fear.	2D motion, audio, color, tilt and height	Lovotics	No	No
2014 [67]	Empathic Robots for Long-Term Interaction	- Affect detection - Empathic appraisal - Action selection - Supportive behaviors - Memory of past interactions	Hoffman theory of empathy, Scherer’s theory, framework of Cutrona et al. [96].	Empathic expressions: Stronger reward, expected reward, weaker reward, unexpected reward, stronger punishment, expected punishment, weaker punishment, unexpected punishment.	Speech, facial expressions Camera	iCat	Yes	No
2014 [79]	An Emotional Model for Social Robots: Late-Breaking Report	- Current task for emotional assessment - Scale for exhaustion - Emotional valence	Reinforcement learning algorithm.	Joy and anger.	Actuators Body postures	-	No	No
2014 [126]	Development of First Social Referencing Skills: Emotional Interaction to Regulate Robot Behavior	- Facial expression recognition - Internal values	Deep learning techniques, attention visual concepts.	Sadness, surprise, happiness, hunger and neutral.	Actuators Camera	Katana arm	No	No
2015 [84]	A Cognitive and Affective Architecture for Social Human–Robot Interaction	CAIO (Cognitive and Affective Interaction-Oriented): - Multimodal perception - Memory - Appraisal - Deliberation - Planning - Multimodal action renderer	Architecture BDI BIGRE mental states.	Regret, disappointment, guilt, reproach, moral satisfaction, admiration, rejoicing, and gratitude.	Actuators (body postures) Camera, microphones	NAO	Yes	No
2015 [32]	Cognitive Emotional Regulation Model in Human–Robot Interaction	- External stimulus emotion from expression - Cognitive reappraisal - Current emotion - Information source in emotional states space	Emotional regulation, based on Gross re-evaluation-based emotional regulation.	Angry, sober, controlled, friendly, clam, dominant painful, interested, humble, excited stiff and influential.	Facial expressions, body postures (head, arms)	-	Yes	No
2015 [61]	The Affective Loop: A Tool for Autonomous and Adaptive Emotional Human-Robot Interaction	Affective loop: - Perception of users’ emotional states - Management of artificial emotions - Learning emotional expressions - Planning of robotic behavior	Definition of empathy by Goleman and Hoffman.	Sadness, anger, disgust, surprise, joy, anger, fear.	Body postures Kinect	NAO	Yes	No
2015 [101]	EMIA: Emotion Model for Intelligent Agent	EMIA: - Perception - Reaction - Encoder - Appraisal - Emotion elicitation - Emotion transition	Fuzzy logic, appraisal theories of emotions, emotion regulation theory, and multistore human memory model.	Happiness, anger, fear, sadness, disgust, and surprise.	-	-	No	No
2016 [102]	A cognitive-affective architecture for ECAs	ECA: - Long-term memories (procedural memory, semantic memory, episodic memory) - Reinforcement learning, chunking, semantic learning - Working memory	Affective model inspired by ALMA.	PAD model.	-	-	No	No
2017 [99]	Towards an Affective Cognitive Architecture for Human–Robot Interaction for the iCub Robot	- Perceiving the state of the human - Before performing an action - After performing an action	Inspired in cognitive architectures.	Neutral, interested, and bored.	-	iCub NAO	No	No
2017 [124]	Animating the Adelino robot with ERIK: the expressive robotics inverse kinematics	ERIK (Expressive Robotics Inverse Kinematics)	Animation and kinematics.	-	Actuators	Adelino	No	No
2018 [64]	SEAI: Social Emotional Artificial Intelligence Based on Damasio’s Theory of Mind	SEAI: - SENSE - PLAN - ACT	Theory of Damasio.	-	Actuators (facial and body expression)	Face robot	No	No
2018 [105]	Multimodal expression of Artificial Emotion in Social Robots Using Color, Motion, and Sound	Expressions of emotions: - Joy is light and warm - Sadness is darkness and blue, low saturation, and reduced brightness - Fear is darkness, black and gray colors - Anger is seeing red, hot fluid	Theory of metaphor and emotion	Joy, sadness, fear, and anger.	Light, motors, and sound.	Probe	No	No
2019 [95]	A multimodal affective computing approach for children companion robots	- Multimodal data - Affective computation - Cognitive computation - Output: dialogue interaction; visual interaction	Three-dimensional space theory PAD, OCC model, RULER Theory.	Happy, angry, and upset.	Text, pronunciation intonation, eye-gaze, gesture, body posture, visual and dialogue interaction	-	Yes	No
2019 [116]	Empathic robot for group learning	- Perception: user awareness, emotional climate, user actions - Memory - Game AI, student modelling, task management - Rapport - Hybrid behavior	Artificial robotic tutors.	-	Body expressions, camera, microphone	Nao Torso	Yes	No
2019 [111]	Artificial emotion modelling in PAD emotional space and human–robot interactive experiment	- Event - Attention filter - Personality - Emotion selection - Evaluate function	PAD emotion space, OCC theory.	Angry, bored, curious, dignified, elated, hungry, inhibited, loved, puzzled, sleepy, violent.	Actuators Camera, microphone	Fuwa	No	No
2020 [81]	An Autonomous Cognitive Empathy Model (ACEM) Responsive to Users’ Facial Emotion Expressions	- Emotion detection - Perspective taking - Empathic behavior provider	Empathy theories	Happiness, sadness, fear, anger, surprise, disgust.	Speech, eye color, motion camera, microphone	Pepper	No	No
2020. [107]	Social and Emotional Skills Training with Embodied Moxie	START Evidence based therapeutic strategies: - Naturalistic applied behavior analysis - Goal-oriented activities - Graded cueing - Cognitive behavior therapy - Adaptive training	ABA (applied behavior analysis) therapy, CBT (cognitive behavioral therapy) for children with ASD.	-	Speech, facial expressions Camera, microphone	Moxie	Yes	Yes
2020 [56]	Deep interaction: wearable robot-assisted emotion communication for enhancing perception and expression ability of children with autism spectrum disorder	- Data collection module - Emotion cognition module - Motion analysis module	Deep learning and multimodal data.	Happiness, anger and fear.	-	-	Yes	Yes
2020 [108]	A Multimodal Emotional Human–Robot Interaction Architecture for Social Robots Engaged in Bidirectional Communication	- Multimodal-affect recognition system - Robot emotion - Robot controller	Multimodal data fusion, OCC model.	happy, interested, sad, worried, and angry.	LEDs, actuators, speech synthesis Kinect, microphone, touch sensor, camera	NAO	No	No
2020 [112]	An affective decision-making model with applications to social robotics	- Affective element (personality, mood, emotion) - Action - Sensors	Based on Gomez and Rios’s affective model for social agent [115].	Hope, fear, joy, sadness, anger.	-	-	No	No
2020 [109]	On Designing Expressive Robot Behavior: The Effect of Affective Cues on Interaction	- Speech recognition - Emotion detection (keywords) - Facial expression - Story comments - Gesture generator	Multimodal data.	Sadness, disgust, happiness, anger, and fear.		ALICE	No	No
2020 [77]	Abel: Integrating Humanoid Body, Emotions, and Time Perception to Investigate Social Interaction and Human Cognition	- Facial expressiveness - PLAN, SENSE, ACT	Extension of SEAI, Damasio’s theory.	-	Facial expressions	ABEL	Yes	Yes
2020 [137]	Creating and capturing artificial emotions in autonomous robots and software agents	ARTEMIS: - Agent Knowledge Graph - Perception - Decision-making or planning - Currently active goal - Goal selection - Action	Scherer theory, PAD model, memory of Dorner’s Psi theory.	Novelty, valence, goal, certainty, urgency goal congruence, coping, norms.	-	-	No	No
2020 [81]	Toward a reinforcement learning-based framework for learning cognitive empathy in human–robot interactions	- Emotion detection - Empathy - Action	Reinforcement learning.	Anger, happiness, and surprise.	Actuators, eye color, speech Camera, microphone	Pepper	No	No
2021 [138]	FATiMA Toolkit: Toward an effective and accessible tool for the development of intelligent virtual agents and social robots	- Integrated authoring tool - Emotional appraisal - Emotional decision-making - Dialogue manager - Reasoners - Role-play character	FATiMA extended.	22 emotions of OCC theory.	-	-	No	No
2021 [82]	Cognitive emotional interaction model of robot based on reinforcement learning	-	Reinforcement learning, PAD model, psychology theory of interpersonal communication.	Happiness, anger, fear, sadness, disgust, and surprise.	-	-	No	No

## 6. Discussion

Several different robots have been proposed for children with ASD, such as Jibo, Cozmo, Keepon, KASPAR, and Zeno. However, it remains unclear how the robots must be designed to express emotions and interact with children. It is necessary that there are design guidelines on how they should be designed, but there have been no studies. Appearance, motion, and expressiveness must also be considered to build intelligent, emotional communication systems. Therefore, designing a social robot requires the collaboration of experts from different disciplines who need to understand each other.

The morphology of robots can help the robot to empathize with children, such as the iCub robot was designed with the appearance of a child. However, in the studies presented, the affective model of this robot was not focused on children with ASD. Breazeal [139] stated that by sharing a similar morphology, robots could communicate in a manner that supports natural communication. Cominelli et al. [77] mentioned that the robot body should be considered when building any abstraction, reasoning, and feeling of what happens. This was supported by other researchers [140], who reported that intelligence could not exist in the form of an abstract algorithm; conversely, it requires a physical instantiation: a body.

Natural interactions between robots and children require that the robot’s behavior depends on the user’s personality. In the studies found for children with ASD, the Moxie robot is based on personal goals, and applies personality quizzes. Studies found it uses the dimensions of personality of the Big Five model [48], which is, among all personality dimensions, the most influential in the robot’s empathic behavior to the user’s personality. Today, the Big Five is used in many virtual agents through the IBM Watson assistant.

Personality has a strong influence on humans’ behaviors, but it is not clear yet how it can affect interactions. Some of the models found consider personality, such as the TAME [66], robot emotion based on PAD [111], affective decision-making model [112], iGrace [91], EMIA [101], cognitive empathy model [63], and ECAs [102]. Bagheri et al. [63] mention that similarity attraction (similar preferences), and complementary principle (complementary behaviors) can influence humans’ behaviors. However, the authors mention that in the literature, similarity attraction has more compelling experimental support. Human–robot interaction is still in its infancy in terms of exploring these biases and relationships, so there is a lack of empirical evidence to help designers understand the perceptions of robot attributes, especially for personality [141].

Alnajjar et al. [142] mention that personalized robot interventions for autistic children have the necessary requirement that the interventions involve repetitive behavioral training and heterogeneity of ASD symptoms among children. Thus, areas in artificial intelligence and robotic technologies can help in performing more frequent assessments. The authors used the NAO robot and designed an autonomous assessment system based on attention cues combined with an enhanced adaptive semi-autonomous interaction based on child interests. The robot’s function was to increase the attention and engagement levels of the child during sessions of therapy.

Children with ASD have difficulties in recognizing and responding to emotions. These children have problems expressing emotions through facial, voice, and body expressions. Therefore, emotion recognition techniques used in children without ASD are different compared to those of a child with ASD. Drimalla et al. [143] investigated imitation and recognition of facial expressions in children with ASD, where they had to use imitation as an alternative to assess facial expression. Interactions with an atypical child compared to a typical child involve fewer expressions.

The research related to the use of robots is limited. Studies have focused more on how the robot should be designed to express emotions in children with ASD, but whether these intelligent, affective models can help interactions last longer has not been evaluated. However, some preliminary studies show that children with ASD prefer a less anthropomorphic appearance of the robot [144].

Modeling empathy in social robots for children with ASD is required. It plays an important role in social interaction and communication. One of the robots to first achieve this aspect of social interaction was Kismet [120], a social robot, developed by MIT, which is a complex agent with many mechanical characteristics, allowing Kismet to express emotions such as disgust, sadness, interest, happiness, and calmness. Kismet was limited in terms of learning ability and socio-affective interactions; nowadays, interest in designing this type of architecture in robots [145] so that they behave empathically toward humans [146] has increased, though attempts have revealed that the scope of this cognitive-affective interaction is limited, and it is difficult to generalize in different contexts. It is important to design these types of interactions with physical robots that are affective so that they can help the child to understand, manage, and recognize each of the emotional states.

**Figure 3:**
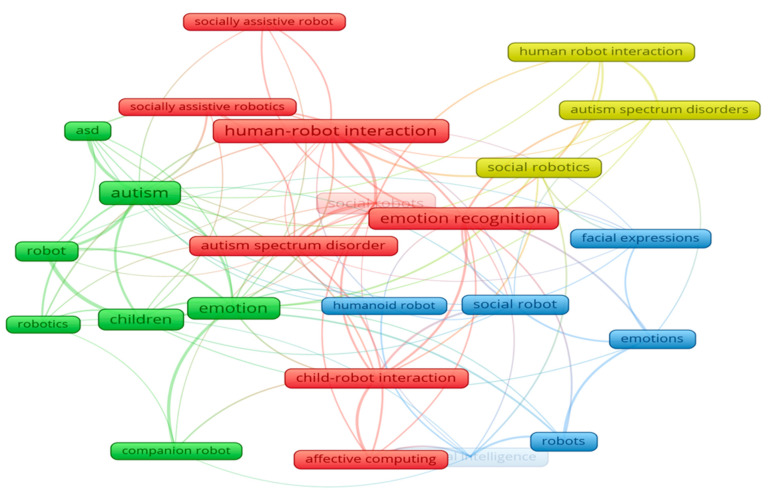
Analysis of keywords cluster (in colors). VOSViewer analyzed the keywords of the selected articles that were used together.

We created a map based on bibliographic data with the keywords “emotions” AND “robots” AND “children” in two databases, WoS and Scopus, using the VOSViewer tool (see Figure 3). Each color represents one cluster, curved lines are relationships associated with the keywords, and density is related to occurrences. The term autism is written as “asd”, “autism”, “autism spectrum disorders”, or “autism spectrum disorder”, and these are related to the four clusters (green, red, yellow, and blue).

The aim was to observe which research areas the studies are most oriented towards. We found that models of emotions for children are more oriented to emotion recognition using facial expressions. However, studies in areas of artificial intelligence are limited. Because the recognition of emotions in children with ASD is still a challenge, they do not express emotions in the same way as a child without ASD, which was observed in the study conducted by [56].

In the selected models of affective communication, we observed that each model was focused on therapy, such as Moxie [98], which focused on ABA therapy, the work proposed by [32], which focused on emotional regulation theories, and Kozima [60], based on the theory of the mind. Thus, there is no definitive comparison of data on which theory is the most effective in embedding these intelligent, emotional communication models.

The model’s inputs may change depending on the communication channels considered by the robot, where outputs may be affected. Cathexis was initially designed to be used for virtual agents. The model was embodied in the Yuppy robot, but it was modified to associate emotional expressions with robot metaphors—another alternative that has not yet been thoroughly evaluated. Thus, the process of designing robotic systems with emotions is different from other areas because many characteristics must be considered, such as autonomous recognition, perception, actions, and their effects on user behavior patterns.

Duquette et al. [147] observed that children with ASD showed greater interest in a robotic partner. The robot was used to model, teach, or practice a skill. Interactions with adults for a child with ASD may not be entirely pleasant; the communication and relationships of an adult with a child compared to a child with another child differ, which has also not been considered in previous studies. The age of the robot may also be a factor affecting a robot’s ability to empathize with children with ASD.

Social robots can be used with children with ASD to capture their attention. Diehl et al. [131] reported that the first task is capturing the user’s attention, then transferring the attention quality of the child to the robot. In this way, SARs can help provide feedback on social interactions between the physical world and children with ASD. The robot can also be a mediator between the therapist and the child. SARs can be used to imitate behaviors.

Boucenna et al. [148] described the contribution of SARs to children with ASD. They mention that social robots are interesting for therapy interventions because robots generate a high degree of motivation in children. Their study emphasized that robots could contribute to the development of social competencies as mimicry and joint attention. However, some concerns are “What should the robot imitate?” and “Which body features would be the most appropriate to achieve this imitation?” So far, most studies have focused on facial expressions. Even imitation involving the arms, face, and head, is complex because there must exist a relationship between them to express an emotional state, including more complex algorithms.

Intelligent, emotional communication models are needed in which the robot learns to react to different participants (children with different severity levels). However, the question is how should these intelligent, emotional communication models be designed? It is unclear that such models and algorithms can be designed and developed for children with ASD, as they cannot be generic models/algorithms. In addition, they must be designed taking into account ethical considerations.

Ojha et al. [10] proposed that robots need to be designed with emotional intelligence, which is why they consider a simplified socio-emotional process as one that can (a) detect social behavior; (b) start a simulation process given said stimulus and allow an internal representation of it; (c) activate an adequate internal visceral emotional state; (d) use past experience and theories to provide an interpretation to the perceived stimulus; (e) adequately regulate the assessment of the emotional state and the expression of appropriate behavior through (c), and other theories related to culture, ethics, morality, and common sense. However, as the authors stated, the studies and models selected for affective social robots or with emotional intelligence use only two of these processes, (a) and (d). Therefore, many challenges remain to be overcome to design these autonomous and child-centered robots with intelligent, emotional communication for children with ASD.

In a review by Cavallo et al. [149], they analyzed emotion models for social robotics, finding a more significant number of studies using the visual channel focused on emotion recognition. The selected studies worked with Ekman [17] or Russell’s theories [19] to express and recognize emotions. However, the use of sensors in vision can be affected by external light sources and whether the person is in front of the camera or in movement. The quality of a vision sensor can affect the quality of signal recognition. However, few studies explored other methods to recognize or express emotions, such as tactile or physiological. Physiological sensors can provide an alternative because they capture direct information at the autonomic nervous system level; however, for children with ASD, this may be invasive. SARs can respond according to the inputs that they receive. Therefore, when the robot captures different signals through the sensors, it captures multimodal data. The interaction with the robot is multimodal through several channels (vision, auditory, gestural, and physiological). Hashimoto et al. [150] describe that for a robot to realize informed interactions with humans, it should be integrating different data that allows it to recognize the environment, such as vision, voice, and gestures. Thus, different input sources in social robots are required. It can help the robot to understand the child’s intentions and emotions, which was indicated by [56], and to recognize emotions in children with ASD.

These data from multiple sources are known as multimodal data. However, with multiple sources that can express the same intention or emotion, appropriate data fusion techniques should be considered to make inferences about the outside world. If these are not applied well, there may be redundancy in the data. Cavallo et al. [149] reported that information obtained is not simply from additional informative channels; the robot can use the information to evaluate the situation and changes in the environment. Few studies have examined the data provided by the sensors to analyze the environment. For example, in [151], fuzzy systems were used to receive information from several channels: sound, temperature, and pressure, to obtain an intelligent, emotional model. However, none of the selected studies mentioned the design of the models, and not all models will work correctly for any given robot, as these models are subject to the inputs that must be evaluated in the environment to create a response.

According to the studies found, intelligent, emotional communication models embodied in social robots for children with ASD must consider some aspects, such as the following:

- The appearance of the physical robot can help it to empathize with the child.
- Communication channels (verbal and non-verbal) to express an appropriate emotional state.
- Types of sensors to perceive emotions, and techniques to recognize a target’s emotion.
- Theories of psychology that can support learning socio-emotional skills.
- Empathic behavior responses are autonomous.

Finally, most of the studies found in social robots research for children with ASD focused on user perception and emotional expressiveness. However, robot learning is still a limited area, and a disconnect exists between perception and the robot’s actions. It is, therefore, necessary to identify the user’s emotions to adapt the robot’s behavior autonomously. Hence, designing a social robot requires the collaboration of experts from different disciplines who need to understand each other.

## 7. Conclusions and Future Work

Affective computing immersed in social robots is limited. Most of the models that have been developed focused on adults, not children, and especially not children with ASD. Affective social robots can provide an alternative to assistive therapy for children with ASD, who have problems related to emotional deficits. We identified that when robots have autonomous cognitive-affective behavior, curiosity is inspired in children. However, it is necessary to correctly use the emotional responses and behavior of a robot to express, recognize, and understand an emotion when interacting with a child with ASD.

Some areas of research that need further study are the artificial intelligence algorithms to build these social intelligence robots for children with ASD. Design guidelines to improve human–robot interaction, how these robots should be designed to empathize with children, and which facial features and body gestures to use should be taken into account for the emotional communication in children with ASD.

Out of the studies selected, only 12 models are focused on children with/without ASD, where three studies are for children with ASD. This shows that the design of these intelligent, emotional communication models is still in preliminary development and the results are not yet clear.

We also observed that in the selected publications, studies of these affective models increased in 2020, which had 10 publications. Among these was the START framework embodied in the Moxie robot, which is focused on children with ASD. However, the authors do not detail the START framework, so it is not clear what flow the model followed. They describe the theoretical basis, but not the AI techniques they use to perceive, express, and respond emotionally. Another aspect that is still preliminary is the evaluation design of these models embodied in social robots for children with ASD, including the number of interventions, the quality of responses, variables of behavior, levels of training, activities, and performance.

Few studies were found on how robots can be integrated for intelligent, emotional communication with children with ASD. Studies were found that applied different theories of psychology, such as emotional regulation and theory of mind. However, there are recommended guidelines on which theories are the most appropriate to build a model centered for children with ASD. It is also unclear if input sensors are more suitable for capturing a child’s emotional behavior, and what output channels may be better to express the emotions. Moxie was the only robot that was found that integrates applied behavior analysis (ABA) and cognitive behavioral therapy (CBT) into the STAR framework. The authors considered two domains: (1) communicating social skills and (2) communicating emotional skills. The communication was focused on non-verbal communication, including facial expressions and gestures. Moxie acts as a companion robot, which increases the interest of the child because they feel that they are not alone.

Emotional communication embodied in robots must be considered in interactions with children with ASD because it motivates children to engage in therapeutic activities. After all, children with ASD have a variety of symptoms and behaviors, as well as severity levels. In addition, the emotions and behaviors of a child may differ compared to those of an adult, especially for children with autism. The response to express emotion that has been most explored is visual, but tactile responses may be an alternative and physiological signal.

Affective computing in human–robot interaction is a subject of interest to researchers due to the advances in emerging technologies. The design of robots that respond autonomously and emotionally may provide an alternative for assistive therapy, especially for children with ASD. However, designing these robots effectively involves perceiving, recognizing, and adapting their behavior in the context of therapy.

No studies were found on robots with imitation skills, which may be an alternative for children with ASD. However, the robot must learn to recognize the child’s behavior and learn to help the child develop and assess the development of socio-emotional skills. The robot must recognize several emotional states when interacting with a child. A database of the child must be created so that the robot can maintain a register of previous interactions and maybe a personality or mood because these can influence social interactions with the child.

Most emotion-focused computational architectures that have been proposed have not been evaluated in physical robots but in virtual agents. Those that have been proposed were oriented more toward cognitive rather than affective architectures. In recent years, there has been an interest in implementing these affective architectures in social robots However, studies still focused more on the design of the robot for children with ASD and on recognizing emotions through multimodal interactions.

For future work, it is necessary to introduce contexts into the interaction. The robot’s behavior will depend on the risk of the child’s condition. Children with ASD do not tolerate surprises, changes in the environment, and so forth. This indicates that social robots must be prepared to adapt to these situations, which could provoke crises in children. In addition, social robots must be equipped with protocols to minimally know whether to change their behavior in the face of crises and the causes that precede them.

## Figures and Tables

**Table 1:** List of keywords used.

Research Keywords
“intelligent” AND “emotions” AND “robots” AND “children”
“intelligent” AND “emotions” AND “robots” AND “autism”
“empathy” AND “robot” AND “children”
“empathy” AND “robot” AND “autism”
“emotional model computational” AND “robots” AND “children”
“affective” AND “architecture” AND “robots” AND “children”

## Data Availability

Not applicable.

## References

[1] Tapus A., Tapus C., Mataric M.J. The use of socially assistive robots in the design of intelligent cognitive therapies for people with dementia. Proceedings of the 2009 IEEE International Conference on Rehabilitation Robotics.

[2] Liu C., Conn K., Sarkar N., Stone W. (2008). Online Affect Detection and Robot Behavior Adaptation for Intervention of Children with Autism. IEEE Trans. Robot..

[3] Eshraghi A.A. (2020). COVID-19: Overcoming the challenges faced by people with autism and their families. Lancet Psychiatry.

[4] American Psychiatric Association (2013). Diagnostic and Statistical Manual of Mental Disorders.

[5] Picard R.W. (2002). Rosalind Picard: Affective Computing. User Model. User-Adapted Interact..

[6] Velásquez J.D., Maes P. (1997). Cathexis: A Computational Model of Emotions. Proceedings of the First International Conference on Autonomous Agents, AGENTS 97.

[7] Velásquez J.D. (1998). When robots weep: Emotional memories and decision-making. American Association for Artificial Intelligence Proceedings.

[8] Esau N., Kleinjohann L., Kleinjohann B. Emotional Communication with the Robot Head MEXI. Proceedings of the 2006 9th International Conference on Control, Automation, Robotics and Vision.

[9] Paiva A., Leite I., Ribeiro T. (2015). Emotion Modeling for Social Robots. The Oxford Handbook of Affective Computing, Psychology Affective Science.

[10] Ojha S., Vitale J., Williams M.-A. (2020). Computational Emotion Models: A Thematic Review. Int. J. Soc. Robot..

[11] Nation K., Penny S. (2008). Sensitivity to eye gaze in autism: Is it normal? Is it automatic? Is it social?. Dev. Psychopathol..

[12] Carter A.S., Davis N.O., Klin A., Volkmar F.R., Volkmar F.R., Paul R., Klin A., Cohen D. (2005). Social Development in Autism. Handbook of Autism and Pervasive Developmental Disorders: Diagnosis, Development, Neurobiology, and Behavior.

[13] Bennett T.A., Szatmari P., Bryson S., Duku E., Vaccarella L., Tuff L. (2013). Theory of Mind, Language and Adaptive Functioning in ASD: A Neuroconstructivist Perspective. J. Can. Acad. Child Adolesc. Psychiatry.

[14] Black M.H., Chen N.T., Iyer K.K., Lipp O.V., Bölte S., Falkmer M., Tan T., Girdler S. (2017). Mechanisms of facial emotion recognition in autism spectrum disorders: Insights from eye tracking and electroencephalography. Neurosci. Biobehav. Rev..

[15] Behrmann M., Thomas C., Humphreys K. (2006). Seeing it differently: Visual processing in autism. Trends Cogn. Sci..

[16] Fox E. (2008). Emotion Science: Cognitive and Neuroscientific Approaches to Understanding Human Emotions.

[17] Ekman P., Friesen W.V., O’Sullivan M., Chan A., Diacoyanni-Tarlatzis I., Heider K., Krause R., Lecompte W.A., Pitcairn T., Ricci-Bitti P.E. (1987). Universals and cultural differences in the judgments of facial expressions of emotion. J. Pers. Soc. Psychol..

[18] Calvo R.A., D’Mello S., Gratch J., Kappas A. (2015). The Oxford Handbook of Affective Computing.

[19] Russell J.A. (1980). A circumplex model of affect. J. Pers. Soc. Psychol..

[20] James W. (1884). What is an Emotion?. Mind.

[21] Lange C.G., James W. (1992). The Emotions.

[22] Schachter S., Singer J. (1962). Cognitive, social, and physiological determinants of emotional state. Psychol. Rev..

[23] Cannon B., Walter B., Brooks C.M., Koizumi K., Pinkston J.O. (1975). Cannon: Personal reminiscences. The Life and Contributions of Walter Bradford Cannon 1871–1945: His Influence on the Development of Physiology in the Twentieth Century.

[24] Lazarus R.S. (1991). Emotion and Adaptation.

[25] Frijda N.H. (1986). The Emotions.

[26] Roseman I.J., Jose P.E., Spindel M.S. (1990). Appraisals of emotion-eliciting events: Testing a theory of discrete emotions. J. Personal. Soc. Psychol..

[27] Ortony A., Clore G., Collins A. (1988). The Cognitive Structure of Emotions.

[28] Damasio A. (1994). Descartes’ Error: Emotion, Reason, and the Human Brain.

[29] Goleman D. (1995). Emotional Intelligence: Why It Can Matter More Than IQ.

[30] Christopher S., Shakila C. (2015). Social Skills in Children with Autism. Indian J. Appl. Res. J..

[31] Gross J.J. (2002). Emotion regulation: Affective, cognitive, and social consequences. Psychophysiology.

[32] Liu X., Xie L., Liu A., Li D. (2015). Cognitive Emotional Regulation Model in Human-Robot Interaction. Discret. Dyn. Nat. Soc..

[33] Gross J.J. (2013). Emotion regulation: Taking stock and moving forward. Emotion.

[34] Salovey P., Mayer J.D. (1990). Emotional Intelligence. Imagin. Cogn. Pers..

[35] Bar-On R. (1997). The Emotional Quotient inventory (EQ-i): A Test of Emotional Intelligence.

[36] Petrides K.V., Furnham A. (2001). Trait emotional intelligence: Psychometric investigation with reference to established trait taxonomies. Eur. J. Pers..

[37] Mayer J.D., Salovey P., Salovey P., Sluyter D. (1997). What is emotional intelligence?. Emotional Development and Emotional Intelligence: Implications for Educators.

[38] Goleman D., Cherniss C., Goleman D. (2001). An EI-based theory of performance. The Emotionally Intelligent Workplace: How to Select for, Measure, and Improve Emotional Intelligence in Individuals, Groups, and Organizations.

[39] Hegel F., Spexard T., Wrede B., Horstmann G., Vogt T. Playing a different imitation game: Interaction with an Empathic Android Robot. Proceedings of the 2006 6th IEEE-RAS International Conference on Humanoid Robots.

[40] Moualla A., Boucenna S., Karaouzene A., Vidal D., Gaussier P. (2018). Is it useful for a robot to visit a museum?. Paladyn J. Behav. Robot..

[41] El-Nasr M.S., Yen J., Ioerger T.R. (2000). FLAME—Fuzzy Logic Adaptive Model of Emotions. Auton. Agents Multi-Agent Syst..

[42] Roseman I.J., Antoniou A.A., Jose P.E. (1996). Appraisal determinants of emotions: Constructing a more accurate and comprehensive theory. Cogn. Emot..

[43] Bolles R.C., Fanselow M.S. (1980). A perceptual-defensive-recuperative model of fear and pain. Behav. Brain Sci..

[44] El-Nasr M.S., Ioerger T., Yen J. PETEEI: A PET with evolving emotional intelligence. Proceedings of the Third International Conference on Autonomous Agents.

[45] Aylett R.S., Louchart S., Dias J., Paiva A., Vala M., Panayiotopoulos T., Gratch J., Aylett R., Ballin D., Olivier P., Rist T. (2005). Fearnot!—An experiment in emergent narrative. Intelligent Virtual Agents.

[46] Gebhard P. (2005). Alma: A layered model of affect. Proceedings of the Fourth International Joint Conference on Autonomous Agents and Multiagent Systems.

[47] Mehrabian A. (1996). Pleasure-arousal-dominance: A general framework for describing and measuring individual differences in temperament. Curr. Psychol..

[48] Digman J.M. (1990). Personality structure: Emergence of the five-factor model. Annu. Rev. Psychol..

[49] Gratch J., Marsella S. (2004). A domain-independent framework for modeling emotion. Cogn. Syst. Res..

[50] Smith C.A., Lazarus R.S. (1990). Emotion and adaptation. Theory and Research, Handbook of Personality.

[51] Yoo S., Jeong O. (2021). EP-Bot: Empathetic Chatbot Using Auto-Growing Knowledge Graph. Comput. Mater. Contin..

[52] Morris C. (2012). The Use of Self-Service Technologies in Stress Management: A Pilot Project. Master’s Thesis.

[53] Laranjo L., Dunn A., Tong H.L., Kocaballi A.B., Chen J., Bashir R., Surian D., Gallego B., Magrabi F., Lau A.Y. (2018). Conversational agents in healthcare: A systematic review. J. Am. Med. Inform. Assoc..

[54] Hoermann S., McCabe K.L., Milne D.N., Calvo R.A. (2017). Application of Synchronous Text-Based Dialogue Systems in Mental Health Interventions: Systematic Review. J. Med. Internet Res..

[55] Moher D., Liberati A., Tetzlaff J., Altman D.G. (2009). The PRISMA Group Preferred Reporting Items for Systematic Reviews and Meta-Analyses: The PRISMA Statement. PLoS Med..

[56] Xiao W., Li M., Chen M., Barnawi A. (2020). Deep interaction: Wearable robot-assisted emotion communication for enhancing perception and expression ability of children with Autism Spectrum Disorders. Future Gener. Comput. Syst..

[57] Chen M., Zhou P., Fortino G. (2016). Emotion Communication System. IEEE Access.

[58] Hirokawa M., Funahashi A., Itoh Y., Suzuki K. Design of affective robot-assisted activity for children with autism spectrum disorders. Proceedings of the 23rd IEEE International Symposium on Robot and Human Interactive Communication.

[59] Shi X.-F., Wang Z.-L., Ping A., Zhang L.-K. (2011). Artificial emotion model based on reinforcement learning mechanism of neural network. J. China Univ. Posts Telecommun..

[60] Kozima H., Nakagawa C., Yano H. (2004). Can a robot empathize with people?. Artif. Life Robot..

[61] Vircikova M., Magyar G., Sincak P., Kim J.H., Yang W., Jo J., Sincak P., Myung H. (2015). The Affective Loop: A Tool for Autonomous and Adaptive Emotional Human-Robot Interaction. Robot Intelligence Technology and Applications 3. Advances in Intelligent Systems and Computing.

[62] Picard R.W. (1995). Affective Computing. Media Laboratory Perceptual Computing Section Technical Report No. 321.

[63] Bagheri E., Esteban P.G., Cao H.-L., De Beir A., Lefeber D., Vanderborght B. (2020). An Autonomous Cognitive Empathy Model Responsive to Users’ Facial Emotion Expressions. ACM Trans. Interact. Intell. Syst..

[64] Cominelli L., Mazzei D., De Rossi D.E. (2018). Social Emotional Artificial Intelligence Based on Damasio’s Theory of Mind. Front. Robot. AI.

[65] Tielman M., Neerincx M., Meyer J.-J., Looije R. Adaptive emotional expression in robot-child interaction. Proceedings of the 2014 9th ACM/IEEE International Conference on Human-Robot Interaction (HRI).

[66] Moshkina L., Park S., Arkin R.C., Lee J.K., Jung H. (2011). TAME: Time-Varying Affective Response for Humanoid Robots. Int. J. Soc. Robot..

[67] Leite I., Castellano G., Pereira A., Martinho C., Paiva A. (2014). Empathic Robots for Long-term Interaction. Int. J. Soc. Robot..

[68] Cañamero L. Modeling motivations and emotions as a basis for intelligent behavior. Proceedings of the 1st International Conference on Autonomous Agents (AGENTS 97).

[69] Gadanho S. (2002). Reinforcement learning in autonomous robots: An empirical investigation of the role of emotions. Emotions in Human and Artifacts.

[70] Murphy R.R., Lisetti C.L., Tardif R., Irish L., Gage A. (2002). Emotion-based control of cooperating heterogeneous mobile robots. IEEE Trans. Robot. Autom..

[71] Ogata T., Sugan S. Emotional Communication Robot: WAMOEBA-2R Emotion Model and Evaluation Experiments. Proceedings of the International Conference on Humanoid Robots.

[72] Velásquez J. Modeling emotions and other motivations in synthetic agents. Proceedings of the Fourteenth National Conference on Artificial Intelligence and Ninth Conference on Innovative Applications of Artificial Intelligence (AAAI’97/IAAI’97).

[73] Izard C.E. (1993). Four Systems for Emotion Activation: Cognitive and Noncognitive Processes. Psychol. Rev..

[74] Goerke N. (2006). EMOBOT: A Robot Control Architecture Based on Emotion-Like Internal Values, Mobile Robotics, Moving Intelligence.

[75] Thomaz A., Berlin M., Breazeal C. An embodied computational model of social referencing. Proceedings of the ROMAN 2005, IEEE International Workshop on Robot and Human Interactive Communication.

[76] Minsky M. (1986). The Society of Mind.

[77] Cominelli L., Hoegen G., De Rossi D. (2021). Abel: Integrating Humanoid Body, Emotions, and Time Perception to Investigate Social Interaction and Human Cognition. Appl. Sci..

[78] Samani H.A., Saadatian E. (2012). A Multidisciplinary Artificial Intelligence Model of an Affective Robot. Int. J. Adv. Robot. Syst..

[79] Truschzinski M., Mïller N. An Emotional Model for Social Robots. Proceedings of the 9th ACM/IEEE International Conference on Human-Robot Interaction (HRI).

[80] Agheri E., Roesler O., Cao H.-L., VanderBorght B. (2020). A Reinforcement Learning Based Cognitive Empathy Framework for Social Robots. Int. J. Soc. Robot..

[81] Bagheri E., Roesler O., Vanderborght B. Toward a Reinforcement Learning Based Framework for Learning Cognitive Empathy in Human-Robot Interactions. Proceedings of the 2020 IEEE/RSJ International Conference on Intelligent Robots and Systems.

[82] Huang H., Li J., Hu M., Tao Y., Kou L. (2021). Cognitive Emotional Interaction Model of Robot Based on Reinforcement Learning. J. Electron. Inf. Technol..

[83] Davis M.H. (2006). Empathy. Handbook of the Sociology of Emotions.

[84] Johal W., Pellier D., Adam C., Fiorino H., Pesty S. (2015). A Cognitive and Affective Architecture for Social Human-Robot Interaction. Proceedings of the Tenth Annual ACM/IEEE International Conference on Human-Robot Interaction Extended Abstracts (HRI’15 Extended Abstracts).

[85] Rao A.S., Georgeff M.P. BDI-agents: From theory to practice. Proceedings of the First International Conference on Multiagent Systems.

[86] Bratman M.E. (1987). Intention, Plans, and Practical Reason.

[87] Davis D., Gwatkin J. (2010). robo-CAMAL: A BDI Motivational Robot. Paladyn J. Behav. Robot..

[88] Hirth J., Berns K., Robots H., Choi B. (2009). Emotion-based Architecture for Social Interactive Robots.

[89] Hobmair H., Altenhan S., Betcher-Ott S., Dirrigl W., Gotthardt W., Ott W. (2003). Psychologie.

[90] Proetzsch M., Luksch T., Berns K. The Behaviour-Based Control Architecture iB2C for Complex Robotic Systems. Proceedings of the German Conference on Artificial Intelligence (KI).

[91] Saint-Aime S., Le Pévédic B., Duhaut D. Children recognize emotions of EmI companion robot. Proceedings of the 2011 IEEE International Conference on Robotics and Biomimetics.

[92] Dang T.H.H., Letellier-Zarshenas S., Duhaut D. GRACE—Generic Robotic Architecture to Create Emotions. Proceedings of the 11th International Conference on Climbing and Walking Robots and the Support Technologies for Mobile Machines—CLAWAR 2008.

[93] Scherer K.R., Dalgleish T., Power M.J. (1999). Appraisal theory. Handbook of Cognition and Emotion.

[94] Isabel B.-M., Mary M. (1985). Manual: A Guide to the Development and Use of the Myers-Briggs Type Indicator.

[95] Chen J., She Y., Zheng M., Shu Y., Wang Y., Xu Y. (2019). A multimodal affective computing approach for children companion robots. Proceedings of the Seventh International Symposium of Chinese CHI (Chinese CHI 19).

[96] Brackett M.A., Bailey C., Hoffmann J.D., Simmons D.N. (2019). RULER: A Theory-Driven, Systemic Approach to Social, Emotional, and Academic Learning. Educ. Psychol..

[97] Hoffman M.L., Bohart A.C., Stipek D.J. (2003). Toward a comprehensive empathy-based theory of prosocial moral development. Constructive & Destructive Behavior: Implications for Family, School, & Society.

[98] Cutrona C., Suhr J., MacFarlane R. (1990). Interpersonal transactions and the psychological sense of support. Personal Relationships and Social Support.

[99] Tanevska A., Rea F., Sandini G., Sciutti A. Towards an Affective Cognitive Architecture for Human-Robot Interaction for the iCub Robot. In Proceeding of the 1st Workshop on Behavior, Emotion and Representation: Building Blocks of Interaction.

[100] Ghiglino D., De Tommaso D., Maggiali M., Parmiggiani A., Wykowska A. (2020). Setup Prototype for Safe Inte Action between a Humanoid Robot (iCub) and Children with Autism-Spectrum Condition. https://osf.io/vk5cm/.

[101] Jain S., Asawa K. (2015). EMIA: Emotion Model for Intelligent Agent. J. Intell. Syst..

[102] Pérez J., Cerezo E., Serón F.J., Rodriguez L.-F. (2016). A cognitive-affective architecture for ECAs. Biol. Inspired Cogn. Arch..

[103] Laird J.E., Wang P., Goertzel B., Franklin S. (2008). Extending the soar cognitive architecture. Frontiers in Artificial Intelligence and Applications.

[104] Ribeiro T., Paiva A. (2017). Animating the adelino robot with ERIK: The expressive robotics inverse kinematics. Proceedings of the 19th ACM International Conference on Multimodal Interaction (ICMI 17).

[105] Löffler D., Schmidt N., Tscharn R. (2018). Multimodal Expression of Artificial Emotion in Social Robots Using Color, Motion and Sound. Proceedings of the 2018 ACM/IEEE International Conference on Human-Robot Interaction (HRI 18).

[106] Kövecses Z. (2003). Metaphor and Emotionâăŕ: Language, Culture, and the Body in Human Feeling.

[107] Hurst N., Clabaugh C., Baynes R., Cohn J., Mitroff D., Scherer S. (2020). Social and Emotional Skills Training with Embodied Moxie. arXiv.

[108] Hong A., Lunscher N., Hu T., Tsuboi Y., Zhang X., Alves S.F.D.R., Nejat G., Benhabib B. (2020). A Multimodal Emotional Human-Robot Interaction Architecture for Social Robots Engaged in Bidirectional Communication. IEEE Trans. Cybern..

[109] Aly A., Tapus A. (2020). On Designing Expressive Robot Behavior: The Effect of Affective Cues on Interaction. SN Comput. Sci..

[110] Ekman P., Friesen W.V. (1987). Facial Action Coding System: A Technique for the Measurement of Facial Movement.

[111] Qingji G., Kai W., Haijuan L. (2008). A Robot Emotion Generation Mechanism Based on PAD Emotion Space. Proceedings of the International Conference on Intelligent Information Processing.

[112] Liu S., Insua D.R. (2019). An affective decision-making model with applications to social robotics. Eur. J. Decis. Process..

[113] Scherer K.R. (2009). Emotions are emergent processes: They require a dynamic computational architecture. Philos. Trans. R. Soc. B Biol. Sci..

[114] Baron-Cohen S., Golan O., Ashwin E. (2009). Can emotion recognition be taught to children with autism spectrum conditions?. Philos. Trans. R. Soc. B Biol. Sci..

[115] Mazzei D., Billeci L., Armato A., Lazzeri N., Cisternino A., Pioggia G., Igliozzi R., Muratori F., Ahluwalia A., De Rossi D. The FACE of autism. Proceedings of the 19th International Symposium in Robot and Human Interactive Communication.

[116] Oliveira P.A., Sequeira P., Melo F.S., Castellano G., Paiva A. (2019). Empathic Robot for Group Learning. ACM Trans. Hum. Robot Interact..

[117] Yu J., Xie L., Wang Z., Xia Y., Sun F., Zhang J., Tan Y., Cao J., Yu W. (2008). An Affective Model Applied in Playmate Robot for Children. Advances in Neural Networks—ISNN 2008.

[118] Feinman S., Roberts D., Hsieh K.F., Sawyer D., Swanson K., Feinman S. (1992). A critical review of social referencing in infancy. Social Referencing and the Social Construction of Reality in Infancy.

[119] Davies M., Stone T., Davies M., Stone T. (1995). Introduction. Folk Psychology: The Theory of Mind Debate.

[120] Young J.E., Hawkins R., Sharlin E. (2008). Igarashi, T. Toward Acceptable Domestic Robots: Applying Insights from Social Psychology. Int. J. Soc. Robot..

[121] Meltzoff A., Heyes B.G.C.M. (1996). The human infant as imitative generalist: A 20-year progress report on infant imitation with implications for comparative psychology. Social Learning in Animals: The Roots of Culture.

[122] Charman T. (2003). Why is joint attention a pivotal skill in autism?. Philos. Trans. R. Soc. B Biol. Sci..

[123] Cibralic S., Kohlhoff J., Wallace N., McMahon C., Eapen V. (2019). A systematic review of emotion regulation in children with Autism Spectrum Disorder. Res. Autism Spectr. Disord..

[124] Mayadunne M.M.M.S., Manawadu U.A., Abeyratne K.R., De Silva P.R.S. A Robotic Companion for Children Diagnosed with Autism Spectrum Disorder. Proceedings of the 2020 International Conference on Image Processing and Robotics (ICIP).

[125] Cañamero L. (2019). Embodied Robot Models for Interdisciplinary Emotion Research. IEEE Trans. Affect. Comput..

[126] Boucenna S., Gaussier P., Hafemeister L. (2013). Development of First Social Referencing Skills: Emotional Interaction as a Way to Regulate Robot Behavior. IEEE Trans. Auton. Ment. Dev..

[127] Suzuki K., Camurri A., Ferrentino P., Hashimoto S. Intelligent agent system for human-robot interaction through artificial emotion. Proceedings of the SMC’98 Conference, 1998 IEEE International Conference on Systems, Man, and Cybernetics (Cat. No. 98CH36218).

[128] Toda M. (1994). The Urge Theory of Emotion and Cognition.

[129] Gadanho S.C., Hallam J. (2001). Robot Learning Driven by Emotions. Adapt. Behav..

[130] Yamaguchi T., Ando N. Intelligenrobot system using “model of knowledge, emotion and intention” and “information sharing architecture”. Proceedings of the IECON’01, 27th Annual Conference of the IEEE Industrial Electronics Society (Cat. No. 37243).

[131] Diehl J.J., Schmitt L.M., Villano M., Crowell C.R. (2012). The clinical use of robots for individuals with Autism Spectrum Disorders: A critical review. Res. Autism Spectr. Disord..

[132] Ahn H.S., Baek Y.M., Na J.H., Choi J.Y. Multi-dimensional emotional engine with personality using intelligent service robot for children. Proceedings of the 2008 International Conference on Control, Automation and Systems.

[133] Watzlawick P., Beavin J.H., Jackson D.D. (2000). Menschliche Kommunikation.

[134] Bartsch K., Wellman H. (1989). Young children’s attribution of action to beliefs and desires. Child Dev..

[135] Luo Q., Zhao A., Zhang H., Wang Y., Li T. (2011). A Layered Model of Artificial Emotion Merging with Attitude. Foundations of Intelligent Systems. Advances in Intelligent and Soft Computing.

[136] Hasson C., Gaussier P., Boucenna S. (2011). Emotions as a dynamical system: The interplay between the meta-control and communication function of emotions. Paladyn J. Behav. Robot..

[137] Hoffmann C., Vidal M.-E. (2020). Creating and Capturing Artificial Emotions in Autonomous Robots and Software Agents. Proceedings of the International Conference on Web Engineering.

[138] Mascarenhas S., Guimaraes M., Santos P.A., Dias J., Prada R., Paiva A. (2021). FAtiMA Toolkit—Toward an effective and accessible tool for the development of intelligent virtual agents and social robots. arXiv.

[139] Breazeal C. (2003). Emotion and sociable humanoid robots. Int. J. Hum. Comput. Stud..

[140] Dautenhahn K., Ogden B., Quick T. (2002). From embodied to socially embedded agents—Implications for interaction-aware robots. Cogn. Syst. Res..

[141] Robert L., Alahmad R., Esterwood C., Kim S., You S., Zhang Q. (2020). A Review of Personality in Human Robot Interactions. https://ssrn.com/abstract=3528496.

[142] Alnajjar F., Cappuccio M., Renawi A., Mubin O., Loo C.K. (2020). Personalized Robot Interventions for Autistic Children: An Automated Methodology for Attention Assessment. Int. J. Soc. Robot..

[143] Drimalla H., Baskow I., Behnia B., Roepke S., Dziobek I. (2021). Imitation and recognition of facial emotions in autism: A computer vision approach. Mol. Autism.

[144] Robins B., Dautenhahn K., Dubowski J. (2006). Does appearance matter in the interaction of children with autism with a humanoid robot?. Interact. Stud..

[145] Rodrigues S., Mascarenhas S., Dias J., Paiva A. “I can feel it too!”: Emergent empathic reactions between synthetic characters. Proceedings of the International Conference on Affective Computing & Intelligent Interaction (ACII).

[146] Toyohashi University of Technology (2015). Humans can empathize with robots: Neurophysiological evidence for human empathy toward robots in perceived pain. ScienceDaily.

[147] Duquette A., Michaud F., Mercier H. (2007). Exploring the use of a mobile robot as an imitation agent with children with low-functioning autism. Auton. Robot..

[148] Boucenna S., Narzisi A., Tilmont E., Muratori F., Pioggia G., Cohen D., Chetouani M. (2014). Interactive Technologies for Autistic Children: A Review. Cogn. Comput..

[149] Cavallo F., Semeraro F., Fiorini L., Magyar G., Sinčák P., Dario P. (2018). Emotion Modelling for Social Robotics Applications: A Review. J. Bionic Eng..

[150] Hashimoto S., Narita S., Kasahara H., Shirai K., Kobayashi T., Takanishi A., Sugano S., Yamaguchi J., Sawada H., Takanobu H. (2002). Humanoid Robots in Waseda University—Hadaly-2 and WABIAN. Auton. Robot..

[151] Salmeron J.L. (2012). Fuzzy cognitive maps for artificial emotions forecasting. Appl. Soft Comput..

